# Decadal–centennial-scale solar-linked climate variations and millennial-scale internal oscillations during the Early Cretaceous

**DOI:** 10.1038/s41598-022-25815-w

**Published:** 2022-12-19

**Authors:** Hitoshi Hasegawa, Nagayoshi Katsuta, Yasushi Muraki, Ulrich Heimhofer, Niiden Ichinnorov, Hirofumi Asahi, Hisao Ando, Koshi Yamamoto, Masafumi Murayama, Tohru Ohta, Masanobu Yamamoto, Masayuki Ikeda, Kohki Ishikawa, Ryusei Kuma, Takashi Hasegawa, Noriko Hasebe, Shoji Nishimoto, Koichi Yamaguchi, Fumio Abe, Ryuji Tada, Takeshi Nakagawa

**Affiliations:** 1 Faculty of Science and Technology, Kochi University, Kochi, 780-8520 Japan; 2 Faculty of Education, Gifu University, Gifu, 501-1193 Japan; 3 Institute for Space–Earth Environmental Research, Nagoya University, Nagoya, 464-8601 Japan; 4 Institute of Geology, Leibniz University Hannover, 30167 Hannover, Germany; 5 Institute of Paleontology, Mongolian Academy of Sciences, Ulaanbaatar, 15160 Mongolia; 6 Center for Advanced Marine Core Research, Kochi University, Kochi, 783-8502 Japan; 7 Faculty of Science, Ibaraki University, Mito, 310-8512 Japan; 8 Nagoya University Museum, Nagoya, 464-8601 Japan; 9 Faculty of Agriculture and Marine Sciences, Kochi University, Kochi, 783-8502 Japan; 10 Faculty of Education and Integrated Arts and Sciences, Waseda University, Tokyo, 169-8050 Japan; 11 Faculty of Environmental Earth Science, Hokkaido University, Sapporo, 060-0810 Japan; 12 Department of Earth and Planetary Science, The University of Tokyo, Tokyo, 113-0033 Japan; 13 Faculty of Earth Science and Civil Engineering, Kanazawa University, Kanazawa, 920-1192 Japan; 14 Institute of Nature and Environmental Technology, Kanazawa University, Kanazawa, 920-1192 Japan; 15 Aichi University, Nagoya, 453-8777 Japan; 16 Nagoya Municipal Industrial Research Institute, Nagoya, 456-0058 Japan; 17 Institute for Geo-Cosmology, Chiba Institute of Technology, Chiba, 275-0016 Japan; 18 Research Centre for Palaeoclimatology, Ritsumeikan University, Kusatsu, 525-8577 Japan

**Keywords:** Palaeoclimate, Projection and prediction

## Abstract

Understanding climate variability and stability under extremely warm ‘greenhouse’ conditions in the past is essential for future climate predictions. However, information on millennial-scale (and shorter) climate variability during such periods is scarce, owing to a lack of suitable high-resolution, deep-time archives. Here we present a continuous record of decadal- to orbital-scale continental climate variability from annually laminated lacustrine deposits formed during the late Early Cretaceous (123–120 Ma: late Barremian–early Aptian) in southeastern Mongolia. Inter-annual changes in lake algal productivity for a 1091-year interval reveal a pronounced solar influence on decadal- to centennial-scale climatic variations (including the ~ 11-year Schwabe cycle). Decadally-resolved Ca/Ti ratios (proxy for evaporation/precipitation changes) for a ~ 355-kyr long interval further indicate millennial-scale (~ 1000–2000-yr) extreme drought events in inner-continental areas of mid-latitude palaeo-Asia during the Cretaceous. Millennial-scale oscillations in Ca/Ti ratio show distinct amplitude modulation (AM) induced by the precession, obliquity and short eccentricity cycles. Similar millennial-scale AM by Milankovitch cycle band was also previously observed in the abrupt climatic oscillations (known as Dansgaard–Oeschger events) in the ‘intermediate glacial’ state of the late Pleistocene, and in their potential analogues in the Jurassic ‘greenhouse’. Our findings indicate that external solar activity forcing was effective on decadal–centennial timescales, whilst the millennial-scale variations were likely amplified by internal process such as changes in deep-water formation strength, even during the Cretaceous ‘greenhouse’ period.

## Introduction

Evidence for millennial-scale (~ 1000–2000 year) climatic oscillations are widely recognized in the paleoclimate records, such as Bond events in the Holocene and Dansgaard-Oeschger (DO) events in the last glacial period^1–6^. The long-term Antarctic ice-core records further reveal that abrupt DO-like oscillations are pronounced during the ‘intermediate glacial’ state (i.e., transition phase between ‘interglacial’ and ‘glacial maximum’)^7,8^, which is likely caused by polar-ice melting and associated changes in the meridional overturning circulation^9^. On the other hand, climatic variations on decadal to centennial timescales^10–13^, and their marked correlation with solar activity changes^14,15^, are also demonstrated in Holocene and late Pleistocene palaeoclimate records. However, studies of decadal- to millennial-scale climatic variations are rare for time intervals prior to the Pleistocene (except for some studies, such as solar-linked cyclicities in the Miocene^16,17^, Eocene^18–20^, Cretaceous^21^, and DO-like oscillations in the Jurrasic^22^), essentially due to the lack of suitable archives capable for such high temporal resolution and longer time-range analysis.

Understanding the behavior of the global climate system during the past extremely warm ‘greenhouse’ periods is essential for predicting ongoing global warming^23,24^. Under the most extreme IPCC (Intergovernmental Panel on Climate Change) projections^25^, atmospheric CO_2_ will surpass 1000 ppm by AD 2100, with a concurrent increase in global surface temperatures of up to 4.5 °C, along with significant loss of polar ice. These conditions are similar to those of the past ‘greenhouse’ periods, such as the mid-Cretaceous (123–90 Ma)^26^, characterized by high atmospheric *p*CO_2_ (ca. 800–1500 ppm)^27^, reduced equator-to-pole temperature gradient^26^, little or no polar-ice^28^, and frequent occurrence of oceanic anoxic events (OAEs)^29^. Modeling studies have also suggested the existence of bi-polar seesaw-type oscillations in ocean circulation during the Aptian–Albian^30^, and centennial- to millennial-scale Arctic temperature variability during the Albian–Turonian^31^, indicating unstable climate conditions. However, the nature of millennial-scale (and shorter) climatic variability and stability under such ‘greenhouse’ conditions remains largely uncertain.

There is a limited number of studies reconstructing millennial-scale climatic variations during the Cretaceous using marine deposits^32,33^. Using marine sediment core, a recent study presented prominent centennial- to millennial-scale variations with possible link with solar activity cycle during the mid-Cenomanian^21^. Marine sediment varve records also document interannual- to decadal-scale climate variability during the Late Cretaceous^34,35^. However, the temporal coverage of the existing studies is insufficient for understanding phenomena occurring on longer time-ranges (i.e., decadal- to millennial-scale variability). Annually laminated lacustrine records^18–20,36–38^ can provide the required high temporal resolution and long-ranging time-series of continental climate variability from the geologic past.

**Figure 1:**
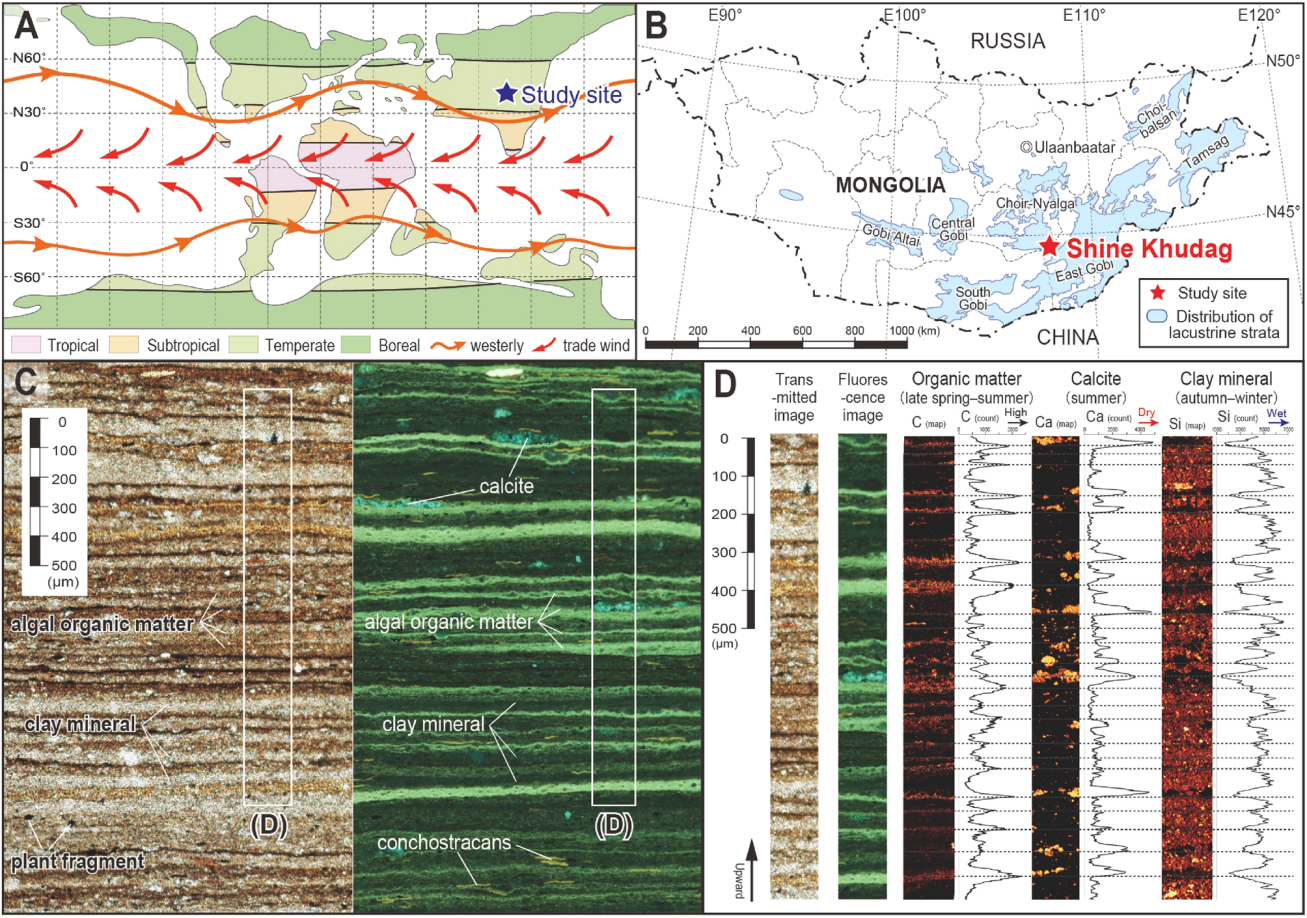
Palaeogeographic map, location, photomicrographs, and laminae compositions of the studied Mongolian lacustrine deposits. (**A**) Reconstructed mid-Cretaceous palaeoclimatic zonation and palaeo-wind patterns^40^. (**B**) Distribution of Lower Cretaceous lacustrine strata and location of study site in Mongolia^39^. (**C**) Photomicrographs of tens of micrometer-scale laminae in shale bed taken under transmitted (left) and reflected fluorescence (right) light. (**D**) Elemental composition of lamina couplets based on SEM–EDX analysis, consisting of micrometer-scale alternations of organic carbon (fluorescent algal OM), lenticular micritic calcite aggregates, and detrital clay mineral (less fluorescent) layers, in ascending order.

Here, we present the evidence for continuous multi-timescale (decadal- to orbital-scale) continental climate variability during the late Early Cretaceous from an annually laminated lacustrine deposit located in southeastern Mongolia (Fig. 1)^39^. Decadal- to centennial-scale variability is obtained from annual lamination (varve) analysis using fluorescence microscopy, while centennial- to orbital-scale variations are reconstructed using ultra-high-resolution XRF scanning.

## Results and discussion

### Solar influence of decadal- to centennial-scale climatic variations

Annually laminated lacustrine deposits of the Shinekhudag Formation are widely distributed in southeastern Mongolia, which was located in the humid temperate climate belt in mid-latitude palaeo-Asia^40^ during the late Early Cretaceous (ca. 123–120 Ma^39^: late Barremian–early Aptian) (Fig. 1A,B, Fig. S1). The deposits consist mainly of decimeter- to meter-scale alternating beds of dark-gray shale, gray dolomitic marl and light-gray dolomite. Shales were deposited in a deep lacustrine basin during lake-level highstands, whereas dolomites were deposited as primary precipitates in a hypersaline environment during lowstand phases^41,42^. These lithologic changes record lake-level fluctuations caused by millennial- to orbital-scale hydrological variations (Figs. S2–S6).

**Figure 2:**
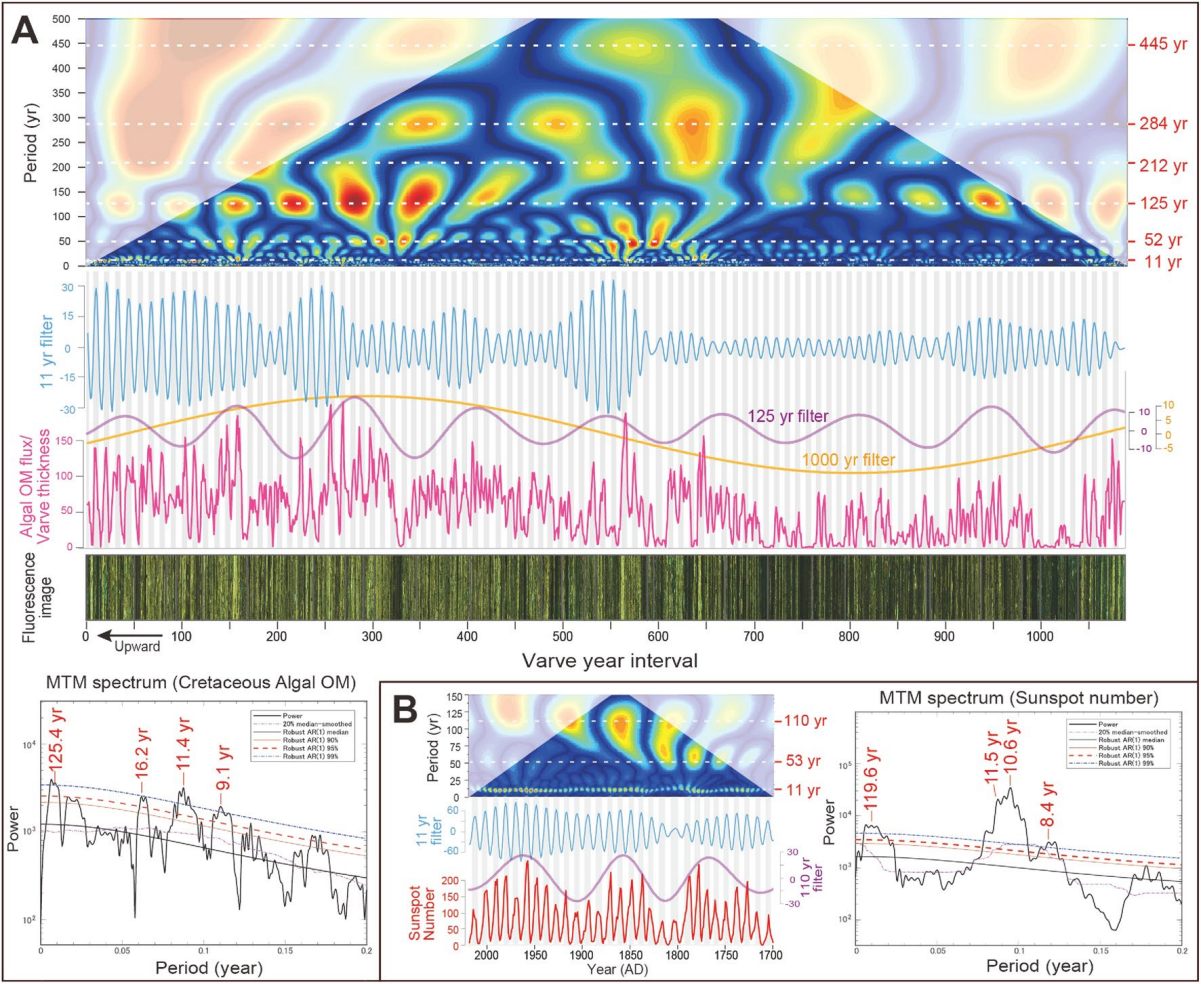
Annually resolved changes in lake algal productivity for a 1091-year interval, compared with modern solar activity. (**A**) Pink line shows the 3-year moving average of changes in algal OM flux, reconstructed from fluorescence intensity and thickness of spring–summer algal OM micro-layers (Figs. S8, S9). Lithology is shown as a reflected fluorescent light image, transformed to temporal variations. The wavelet analysis, MTM spectrum (for 0–500 varve year interval), and AM of 11-year filter (light blue; frequency: 0.089; bandwidth: 0.01), 125-year filter (purple; frequency: 0.008; bandwidth: 0.002), and 1000-year (yellow; frequency: 0.001; bandwidth: 0.0002) filters are also shown. Reddish numbers in MTM spectrum indicate significant spectral peaks above 99% confidential level (CL). Gray vertical bars highlight correlations between changes in lake algal productivity and 11-year cycles. (**B**) Changes in sunspot activity for the past 320 years, with wavelet analysis, 11-year filter (light blue; frequency: 0.0925; bandwidth: 0.01) and 110-year filter (purple; frequency: 0.0091; bandwidth: 0.005), and MTM spectral results.

Annual laminations are well preserved in the shale beds of the Shinekhudag Formation (Figs. 1C,D, 2A). Fluorescence microscopy and SEM–EDX analyses reveal microfacies that consist of tens of micrometer-scale lamina couplets of strongly fluorescent amorphous organic matter (OM) with lenticular micritic calcite aggregates, and less-fluorescent detrital clay minerals (Fig. 1C,D). The average thickness of laminae is 40–80 μm in shale, and about 100–160 μm in dolomitic marl facies (Fig. S7), which correspond to between 4 and 16 cm/kyr assuming they are annual. This value is in excellent agreement with mean sedimentation rates of ~ 8.3 cm/kyr estimated from orbital cyclicity (see “Methods” section; Figs. S2–S6), and consistent with chronologically calculated rates (between 4.7 ± 2.6 and 10.0 ± 7.6 cm/kyr) based on radiometric dating of intercalated tuffs (Fig. S1D)^39^. Thus, the μm-scale lamina couplets in shale beds of the Shinekhudag Formation are interpreted as lacustrine varves, reflecting seasonal variability.

These lamina couplets resemble the sediment composition of modern carbonate lakes in Minnesota, USA^43^ and northeastern Germany^44,45^. Both modern mid-latitude lakes are characterized by deposits composed of alternations of annually laminated diatom and algal OM, endogenic calcites, and allochthonous clays. These compositional variations reflect seasonal changes in primary productivity, carbonate saturation state of lake surface waters, and terrigenous clay input. Comparison with modern organic-calcite varve analogs^43–45^ suggests that the lamina couplets reflect the deposition of algal OM and biochemically precipitated endogenic calcite in the lake epilimnion during late spring–summer, and clay-dominated deposition during the low-productivity autumn–winter period (Fig. 1D). Accordingly, we assigned algal OM flux (multiplication of fluorescence intensity and thickness of algal OM micro-layer) to reflect algal productivity changes in the lake surface waters during late spring–summer.

Based on the microscopic observation of varve laminae and calculation of algal OM flux, we reconstructed changes in algal productivity for a 1091-year interval (5.5 cm section) (Fig. 2A, Fig. S8). For quantitatively reconstructed changes in algal OM flux, we also used a lamination tracing and unfolding program^46^, which was developed to convert the original ‘folded’ lamination pattern into a straight ‘unfolded’ lamination (Fig. S9). Wavelet analysis and Multi-taper method (MTM) spectrum of the algal productivity variations show marked periodicities of ~ 9.1, 11.4, 16.2, 52, 125, 212, 284, and 445 years, along with longer ~ 1000-year modulation (Fig. 2A). The obtained periodicities of ~ 11, 125, 212, and 445 years correspond to well-documented solar activity cycles during the Holocene, including the 11-year Schwabe, the ~ 88–120-year Gleissberg, the ~ 208-year de Vries cycles, and unnamed ~ 350–500 year cycles^14,15,47–49^ (Table 1). In particular, changes in algal productivity appear to reflect a pronounced ~ 11-year Schwabe cycle, modulated by a ~ 125-year cycle, resembling the modern sunspot cycle (Fig. 2B). Although the obtained ~ 125-year cycle in Cretaceous lake record is slightly different from ~ 110-year cycle of modern sunspot cycle, previous studies pointed that Gleissberg cycle is not a cycle in the strict periodic sense but rather a modulation of the cycle with a varying timescale of ~ 88–120-years (periodicities around 104 yr and 150 yr are also obtained)^47–49^.

**Table 1:** Comparison of multiple frequency (decadal- to millennial-scale) periodicities identified in the geological records.

Record	Age	Decadal- to centennial-scale periodicities (year cycle)							References
Total solar irradiance (TSI), ^14^C, ^10^Be	Holocene	10.9 Schwabe	88–120 Gleissberg	208 de Vries	–	350	506	710	Steinhilber et al.^15^, Abreu et al.^48^ and Usoskin^49^
Hulu Cave and GRIP d^18^O	Late Pleistocene	–	–	–	286	352	512	712	Clemens^64^
Antarctic ice-core EDC δ^18^O	Middle–Late Pleistocene	–	–	–	–	–	–	–	Calculated by this study; data from Barker et al.^7^
Lacustrine deposits Ostracod, GR, MS	Late Miocene	–	110–123	209	–	352	510	–	Kern et al.^16,17^
Lacustrine varve	Middle Eocene	10–11	70–90	–	–	–	–	–	Shi et al.^20^
Lacustrine varve Pollen	Early Eocene	10–11	82	210–231	–	420–438	–	–	Lenz et al.^18,19^
Marine marlstone XRF core scanner	early Late Cretaceous (mid-Cenomanian)	–	80–100	200–230	–	393–412	569	–	Ma et al.^21^
Marine deposits benthic Foraminifera	late Early Cretaceous (Albian OAE1b)	–	–	–	–	–	~ 500	–	Friedrich et al.^32^
Lacustrine varve, XRF core scanner, Ca/Ti variations	late Early Cretaceous (Barremian–Aptian)	11.4	84–125	185–212	263–284	361–412	524–568	686–716	This study
Marine marl-limestone Rock magnetic	Late Jurassic	–	–	–	–	–	–	–	Boulila et al.^22^
Rock magnetic	Early Triassic	–	–	–	–	–	–	–	Wu et al.^60^
Rhythmite	Late Carboniferous–Early Permian	–	–	–	–	–	–	–	Franco et al.^59^
Ti variations	Early Devonian	–	–	–	–	–	–	–	da Silva et al.^61^

Record	Age	Millennial-scale Periodicities (kyr cycle)							References
Total solar irradiance (TSI), ^14^C, ^10^Be	Holocene	0.9–1.2 Eddy	–	2.0–2.5 Hallstatt	–	5.0	–	–	Steinhilber et al.^15^, Abreu et al.^48^ and Usoskin^49^
Hulu Cave and GRIP d^18^O	Late Pleistocene	1.1–1.3	1.5–1.7	2.0–2.2	3.0	4.0–4.8	–	10.0	Clemens^64^
Antarctic ice-core EDC δ^18^O	Middle–Late Pleistocene	–	1.5	2.0–2.5	3.1–3.7	4.5–5.5	7.0–7.4	10.0–11.0	Calculated by this study; data from Barker et al.^7^
Lacustrine deposits Ostracod, GR, MS	Late Miocene	1.0	–	2.3	–	–	–	–	Kern et al.^16,17^
Lacustrine varve	Middle Eocene	–	–	–	–	–	–	–	Shi et al.^20^
Lacustrine varve Pollen	Early Eocene	0.9	1.6–1.7	2.3–2.4	3.6–3.7	–	7.4–7.6	–	Lenz et al.^18,19^
Marine marlstone XRF core scanner	early Late Cretaceous (mid-Cenomanian)	–	1.6–1.7	–	–	~ 4.8	–	–	Ma et al.^21^
Marine deposits Benthic Foraminifera	late Early Cretaceous (Albian OAE1b)	1.25	–	–	–	5.7	–	–	Friedrich et al.^32^
Lacustrine varve, XRF core scanner, Ca/Ti variations	late Early Cretaceous (Barremian–Aptian)	1.0–1.25	1.4–1.5	1.8–2.1	3.2–3.9	4.6–5.6	~ 7.3	10.0–12.0	This study
Marine marl-limestone Rock magnetic	Late Jurassic	–	1.35–1.54	–	–	5.2	–	10.5	Boulila et al.^22^
Rock magnetic	Early Triassic	–	–	–	3.8	4.7–5.3	6.8	11.0	Wu et al.^60^
Rhythmite	Late Carboniferous–Early Permian	–	–	2.0–2.5	3.5–3.6	4.0–5.4	–	11.1–13.6	Franco et al.^59^
Ti variations	Early Devonian	0.8–1.1	–	2.5	–	–	6.0–7.0	10.0–12.0	da Silva et al.^61^

**Figure 3:**
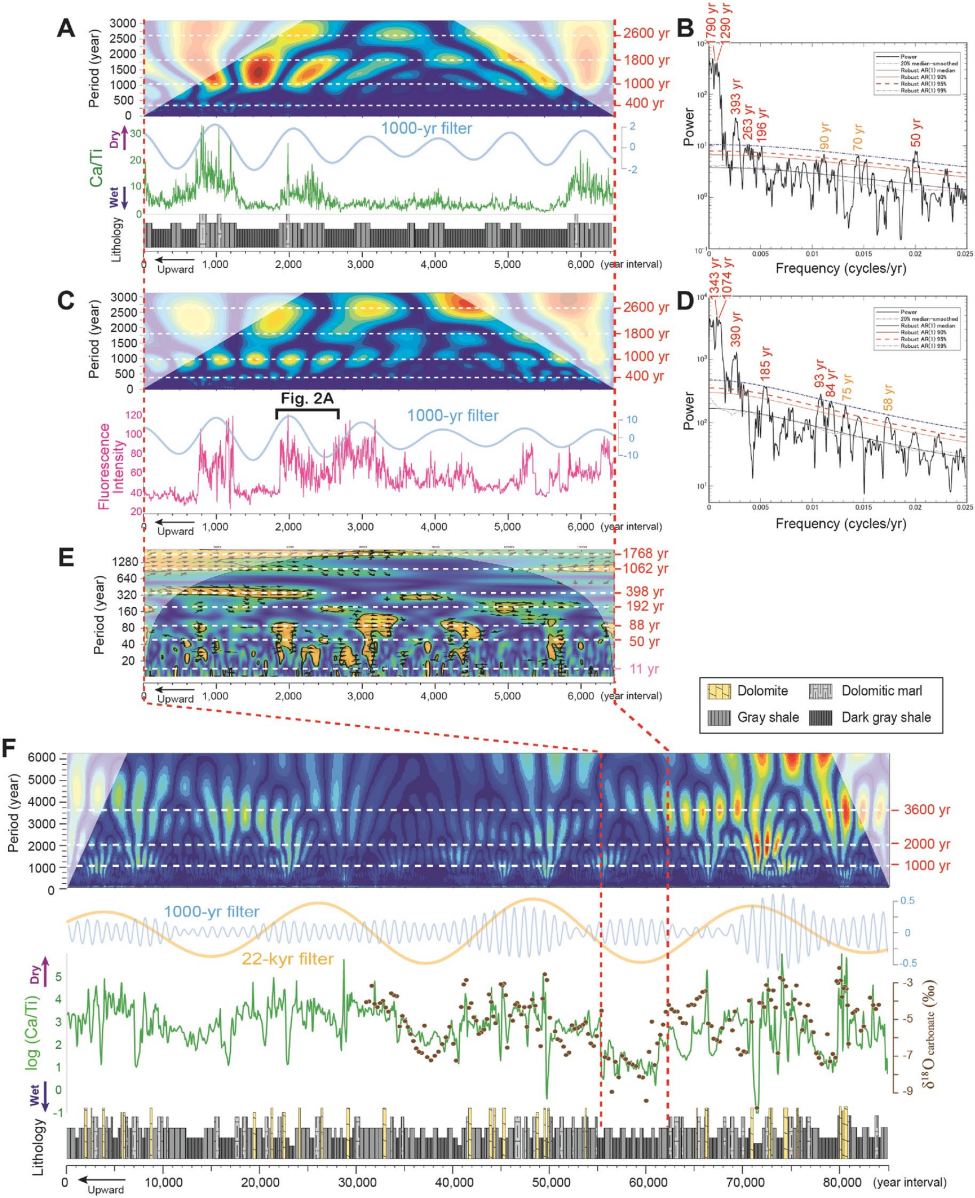
Comparison between changes in Ca/Ti ratios (evaporation/precipitation proxy) and fluorescence intensity of algal OM. (**A,B**) Lithology, Ca/Ti ratios, wavelet analysis, and MTM spectrum for a 55 cm-thick (~ 6.4-kyr) interval of core CSH01 at 60 μm (~ 1 year) resolution using a scanning X-ray analytical microscopy (Horiba, XGT-5000). (**C,D**) Fluorescence intensity of Algal OM, wavelet analysis, and MTM spectrum for a 55 cm-thick (~ 6.4-kyr) interval at 60 μm (~ 1 year) resolution. Reddish and yellowish numbers in MTM spectrum indicate significant spectral peaks above 99% CL and 95% CL, respectively. Amplitude modulation of 1000-year filter (light blue; frequency: 0.001; bandwidth: 0.0002) are also shown. (**E**) Wavelet coherence analysis power spectrum of Ca/Ti ratio and Fluorescence intensity for a 55 cm-thick (~ 6.4-kyr) interval. (**F**) Lithology, Ca/Ti ratios and wavelet analysis for a 7.6-m thick (~ 85-kyr) interval at 500 μm (~ 6 year) resolution using a μ-XRF core scanner (Cox, Itrax). Amplitude modulation of 1000-year filter (light blue; frequency: 9.5e4; bandwidth: 8.0e-5) and 22-kyr filter (yellow; frequency: 4.5e-5; bandwidth: 1.0e-5), and stable oxygen isotopic values of bulk carbonate (brown circle; 2.5 cm spacing, ~ 300-year resolution) are also shown.

**Figure 4:**
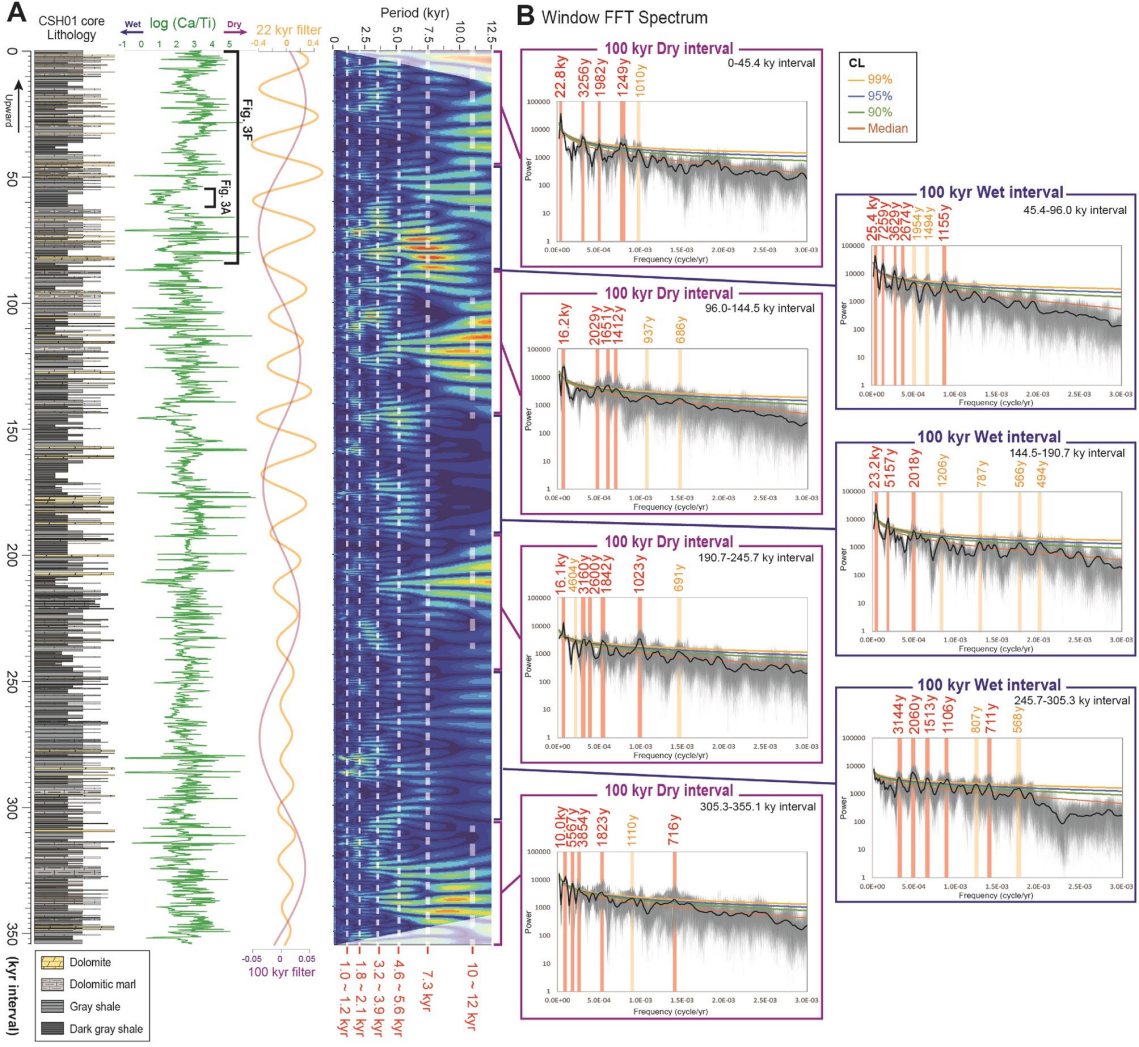
Decadally resolved changes in Ca/Ti ratios for a ~ 355-kyr interval. (**A**) Lithology, Ca/Ti ratios and wavelet analysis for a 29.6 m-thick (~ 355-kyr) interval of core CSH01 at 500 μm (~ 6 year) resolution using a XRF core scanner (Cox, Itrax). Amplitude modulation of 22-kyr filter (yellow; frequency: 4.5e-5; bandwidth: 1.0e-5) and 100-kyr filter (purple; frequency: 9.5e-6; bandwidth: 1.5e-6) are also shown. (**B**) Window FFT analyses of Ca/Ti variations for the drier interval and wetter interval of 100-kyr eccentricity minimum/maximum. Results of the Monte Carlo 1000 random runs concerning uncertainties of relative age estimations (Fig. S7) are shown as semitransparent grey lines. Significances of Fourier power spectrum are estimated for median values (black thick lines) only, assuming that subject power spectrum contains red-noise back ground signal. Reddish bars and numbers indicate significant spectral peaks above 99% CL. Yellowish bars and numbers indicate moderate spectral peaks above 90% CL. Window FFT analysis reveals pronounced but quasi-periodic millennial-scale periodicities centering on ~ 1010–1023, 1106–1155, 1206–1249, 1412–1513, 1823–2060, 3160–3854, 4604–5567, and ~ 7259 years, and less-pronounced centennial-scale periodicities of ~ 566–568, 686–716, and 787–810 years.

In order to obtain information on climatic variations on longer timescales, we analyzed the elemental composition changes of the alternating beds of shale and dolomite using μ-XRF scanning (Figs. 3, 4). A 55 cm-thick section dominated by shale bed (corresponding to a ~ 6.4-kyrs interval) was analyzed at 60 μm spacing (~ 1 year resolution) using a X-ray analytical microscope (Fig. 3A, Fig. S10). Changes in fluorescence intensity of algal OM laminae for the same interval were also analyzed by fluorescence imaging microscopy (Fig. 3C, Fig. S10). A 29.6 m-long section of CSH01 core (corresponding to ~ 355-kyr interval) was analyzed using a XRF core scanner at 500 μm spacing (~ 6-year resolution) (Figs. 3F, 4A, Figs. S5, S6). Ca/Ti ratios show a marked correspondence with lithology, with calcium concentrations reflecting dolomite precipitation in a shallow hypersaline lake^41,42^, whereas Ti concentrations reflect terrigenous clay input to the deep lake. During lake-level highstands, enhanced algal productivity and high terrigenous clay input results in formation of organic-clastic varve. In addition, biochemically-induced calcite precipitation triggered by algal photosynthesis and increased pH of the lake epilimnion result in forming micritic calcite within organic-rich laminae. On the other hand, in response to decreasing lake level, lake epilimnion becomes oversaturated with CaCO_3_, and precipitation of calcite is enhanced. If drier climatic condition is pronounced, increasing salinity (decreasing lake level) and precipitation of calcium carbonate result in an increase in the Mg/Ca ratio in lake water. Then, high Mg/Ca ratio in the arid saline lakes results in deposition of dolomite and high-Mg calcite in their sediments. Therefore, carbonate composition and concentrations in lake sediments as well as flux of terrigenous clay input should be a sensitive indicator of climatically induced changes in the balance between evaporation and precipitation^43^. Thus, the Ca/Ti ratio is taken as a proxy for evaporation/precipitation, with the occurrence of dolomite beds indicating drier events.

Both the fluorescence intensity of algal OM and Ca/Ti ratios for the ~ 6.4-kyrs interval show marked millennial-scale variations (~ 1000–1300, 1800, and 2600 years periodicities), with stronger fluorescence intensities corresponding to drier periods (Fig. 3A,C). Although decadal- to centennial-scale variations are not pronounced compared to millennial-scale, wavelet coherence analysis and Fourier analysis reveal that both changes in algal OM and Ca/Ti are coherent at periodicities of ~ 50–58, 84–93, 185–196, 263, and 390–398 years (Fig. 3B,D,E). The obtained periodicities correspond to the solar cycles of ~ 88–120-year Gleissberg, the ~ 208-year de Vries, unnamed ~ 350–500 year cycles, and the ~ 1000-year Eddy cycles^14,15,47–49^ (Table 1). Therefore, both algal productivity (Figs. 2A, 3C) and evaporation/precipitation changes (Fig. 3A) independently indicate decadal- to millennial-scale periodicities recorded in the Mongolian lake deposits, which correspond to well-documented solar activity changes.

Records from the Holocene and the last glacial demonstrate a clear link between solar activity and decadal–centennial climate change^10–15,47–52^, although some inconsistencies exist^53^. Our findings confirm that solar influence on decadal–centennial climatic variations (including the 11.4-year Schwabe cycle) also existed during the Early Cretaceous. Together with convincing evidence of the 10.6-year cycle from a Permian (~ 291 Ma) fossil tree-ring record^54^, and numbers of evidence of the ~ 11 year Schwabe cycle from lacustrine and marine varve records including Neoproterozoic^55^, the Cretaceous lacustrine archive supports the continuity of the solar dynamo periodicity through geological time. Since intervals of stronger fluorescence intensity in algal OM correspond to drier periods as indicated by higher Ca and lower Ti concentrations (Fig. S10), decadal- to centennial-scale changes in algal productivity were thought to be controlled by regional insolation changes, modulated by variations in regional cloudiness and hydrology (algal photosynthesis is enhanced due to low cloud cover during drier condition), similar to modern lake analogues^44^. Given that the descending limb of the subtropical Hadley circulation was shifted towards lower latitudes during the mid-Cretaceous (Fig. 1A)^40^, variations in regional cloudiness and hydrology at the study site likely reflect changes in moisture transport via westerly winds in mid-latitude palaeo-Asia. This idea is consistent with the possible link between solar activity and mid-latitude westerly jet and storm tracks through the so-called ‘solar top-down mechanism’ in the Holocene record^10–14,50–52^.

### Millennial-scale climate oscillations possibly amplified by internal process

**Figure 5:**
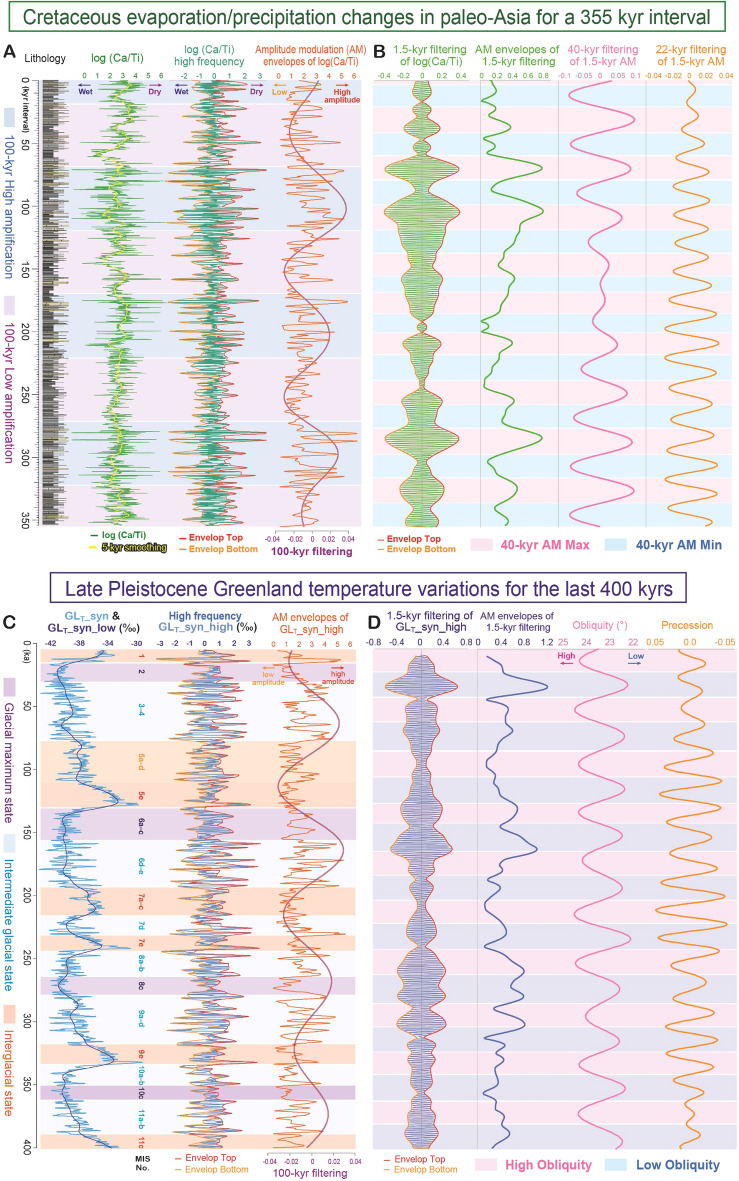
Comparison of millennial-scale variation patterns between the Cretaceous lacustrine deposits (**A,B**) and the late Pleistocene Greenland temperature record (**C,D**). (**A**) Lithology, log (Ca/Ti) with 5-kyr smoothing, high frequency (< 5-kyr) variations of log (Ca/Ti) with envelop top and bottom (2.5-kyr sliding windows), and AM envelopes (subtraction of envelop top and bottom) of log (Ca/Ti) high frequency with its AM of 100-kyr filter (purple; frequency: 9.5e-6; bandwidth: 5.0e-6) for a ~ 355-kyr interval of CSH01 core. (**B**) 1.5-kyr filtering (frequency: 6.7e-4; bandwidth: 4.0e-5) of log (Ca/Ti) with envelop top and bottom (2.5-kyr sliding windows), AM envelopes of 1.5-kyr filtering of log (Ca/Ti), 40-kyr filtering (frequency 2.5e-5; bandwidth: 2.0e-5) and 22-kyr filtering (frequency 4.5e-5; bandwidth: 1.0e-5) of 1.5-kyr AM of log (Ca/Ti). (**C**) Greenland temperature variability record (GL_T__syn and GL_T__syn_high)^7^ of the last 400 kyrs. AM envelopes (2.5-kyr sliding windows) of GL_T__syn_high with its AM of 100-kyr filter (purple; frequency: 1.0e-5; bandwidth: 2.0e-6) are also shown. (**D**) 1.5-kyr filtering (frequency: 6.7e-4; bandwidth: 4.0e-5) of GL_T__syn_high, AM envelopes (2.5-kyr sliding windows) of 1.5-kyr filtering, and obliquity and precession cycles for last 400 kyrs. Note that abrupt millennial-scale oscillations in both Cretaceous lacustrine Ca/Ti ratio and Greenland temperature variability show distinct AM by the obliquity and eccentricity cycle band (see also Boulila et al.^22^ for AM of DO-scale variability of half-precession and precession cycle bands during the last glacial and the Late Jurassic periods).

Long-ranging variations in Ca/Ti ratios further reveal marked millennial-scale oscillations with amplitude modulation (AM) by the ~ 22-kyr precession and ~ 100-kyr short eccentricity cycles (Figs. 3F, 4A, 5A). High Ca/Ti values are associated with dolomite layers, covering ~ 200–400 years each, and show pronounced millennial-scale (~ 1000–2000-year) periodicities (Fig. 3F). The observed amplitude of millennial-scale variations is clearly larger than that of the precession and eccentricity cycles (Fig. 4A). In contrast to the organic-calcite varves that occur in Holocene deep lakes under humid climate conditions^43–45^, lacustrine primary dolomite generally forms in shallow hypersaline lakes under semi-arid climate conditions^41,42^ (Fig. S11, Table S1). Stable oxygen isotopes of bulk rock samples (2.5 cm spacing; ~ 300-year resolution) reveal marked correspondence with Ca/Ti ratio as well as lithofacies, with very negative values (between − 10 and − 7‰) in dark-grey shale layer, while less negative values (between − 4 and − 2‰) in dolomite layer (Fig. 3F). Because of the lack of replacement textures, micro-crystalline dolomite in the deposits is thought to represent autochthonous and primary precipitation from the lake water or early diagenetically from pore water^39^. Such large variations between primary dolomite and shale layers (> 8‰) supports drastic changes in the evaporation/precipitation balance^56^. Thus, the periodic occurrence of dolomite beds in the Shinekhudag Formation likely reflects millennial-scale extreme drought events.

Wavelet analysis and MTM spectrum reveal that the observed millennial-scale drought events are characterized by multiple frequency patterns, with quasi-periodic patterns centering on ~ 1010–1023, 1106–1155, 1206–1249, 1412–1513, 1823–2060, 3160–3854, 4604–5567, and ~ 7259 years (Fig. 4B, Fig. S12). These time-series analysis is performed using a Monte Carlo 1000 random runs concerning uncertainties of relative age estimates (see “Methods” section). Such multiple frequency patterns are persistently observed in both, drier and wetter intervals of the ~ 100-kyr short eccentricity cycle (Fig. 4B). Although the obtained periodicities show similarities with reported solar activity changes of the ~ 1000-year Eddy and ~ 2300-year Hallstatt cycles^14,15,47–49^, the Cretaceous lacustrine record seems more complicated and quasi-periodic.

Multiple frequency patterns of millennial-scale periodicities are also identified in other geological periods^18,19,21,22,57–61^ (Table 1), including the well-documented ‘ ~ 1470-yr cycle’ of Bond events in the Holocene and the DO events in last glacial^1–6^, and the ~ 4500–6000-yr periodicities of Heinrich events^62^. A recent study also pointed out existence of DO-like ~ 1.5-kyr oscillations with AM by half-precession, precession and short eccentricity cycles during the Late Jurassic^22^. Millennial-scale (~ 1000–2000-yr) climatic variability has been linked to either internal (ice sheet dynamics, changes in the ocean–atmosphere system) or external mechanisms (solar forcing or combination tones of orbital-scale cyclicity). Since millennial-scale variability has been observed by previous studies in different palaeoceanographic, palaeogeographic, and palaeoclimatic settings and over time periods from the Devonian to the Quaternary^18,19,21,22,57–61^, a persistent external pacing scenario may not be completely ruled out. Although some previous studies have suggested a possible link between the 1470-year periodicities of Bond and DO events and the centennial-scale solar activity cycle^63,64^, such periodicities are not observed in total solar irradiance. In consequence, Bond and DO events were mostly linked to changes in oceanic circulation and not with variations in solar output^3,5,6,65^. Internally driven oscillating mechanisms are likely involved in controlling millennial-scale climatic variability.

We note that millennial-scale (< 5-kyr) oscillations in Ca/Ti ratio in the Cretaceous palaeo-Asia show distinct AM induced by the 100-kyr short eccentricity cycle (Fig. 5A, Fig. S14A). Moreover, AM of ~ 1.5-kyr band-pass filtering of log (Ca/Ti) data shows the pronounced 40-kyr obliquity cycle (Fig. 5B, Fig. S14B). Interestingly, similar millennial-scale AM induced by Milankovitch cycle band (including, precession, obliquity and eccentricity) was also observed in the abrupt DO climatic oscillations in the late Pleistocene Greenland temperature variability record (GL_T__syn_high)^7^ (Fig. 5C,D, Fig. S14C,D; see also AM analysis for ~ 1.5-kyr filtering of DO events by previous studies^22,66–68^). Furthermore, AM of DO-like ~ 1.5-kyr periodicities modulated by half-precession, precession, and short eccentricity cycles are also observed in the low-latitude marine record of the Late Jurassic^22^. These lines of evidence suggest that AM of ~ 1.5-kyr periodicities induced by the Milankovitch cycle band have occurred in several epochs, both during the ‘icehouse’ and ‘greenhouse’ periods.

Given the presence of half-precession signal in the DO record^69^ and in their potential analogues in the Jurassic^22^, there is debate on the potential low-latitude climate forcing of the ~ 1.5-kyr cycle. The half-precession and precession signals are also observed in late Pleistocene millennial-scale variability records of the African monsoon^69^ and Asian monsoon^70^ regions, although some inconsistencies exist^71^. As discussed in Boulila et al.^22^, the pronounced half-precession signal in the Jurassic can be attributed either to the low-latitude location of the record or to large land-mass of Jurassic continental configuration (involving a large latitudinal shift in the intertropical convergence zone). On the other hand, other studies^66–68^ pointed out that the large AM of the DO ~ 1.5-kyr periodicities in the Greenland region tend to occur during the decreasing phase of obliquity in the late Pleistocene, suggesting a mid- to high-latitude influence (Fig. 5D). It is an intriguing similarity that predominance of obliquity signal in the AM of ~ 1.5 kyr periodicities in the Cretaceous record as well (Fig. 5B, Fig. S14B), although further study is required to understand the causal mechanisms of the AM of the 1.5-kyr band in the ‘greenhouse’ condition.

It is also noteworthy that millennial-scale oscillations in both the Cretaceous Ca/Ti ratios and the late Pleistocene Greenland temperature variability^7^ show distinct AM induced by 100-kyr eccentricity cycle (Fig. 5A,C, Fig. S13). Previous studies have suggested that abrupt millennial-scale DO oscillations occurring in the ‘intermediate glacial’ state (transitional stage of glacial-interglacial cycle) were linked to intermediate size of polar ice-sheets and associated changes in the strength of deep-ocean circulation (i.e., bi-polar seesaw)^7–9^. Our findings indicate that, despite the differences in size of polar-ice-sheets and land–ocean distribution compared with the late Pleistocene, millennial-scale climatic oscillations induced by the short eccentricity cycle (as well as obliquity-paced ~ 1.5-kyr periodicities) also prevailed during the Cretaceous (Fig. 5, Fig. S13).

The marked similarity in millennial-scale oscillations during both the Cretaceous ‘greenhouse’ and late Pleistocene ‘intermediate glacial’ states implies a specific mechanism that enhanced the sensitivity to millennial-scale oscillations under warmer ‘greenhouse’ conditions. The ~ 1000–2000-year oscillations correspond to timescales of deep-ocean circulation, suggesting a possible link with changes in ocean circulation dynamics. Although evidence of millennial-scale variability during the Cretaceous is limited, benthic foraminiferal assemblages^32^ suggest the existence of millennial-scale (~ 1.25 and 5.7 kyr) oscillations in the strength of ocean circulation/ventilation during the Albian OAE1b. A recent study of marine deposits also indicates the existence of millennial-scale (~ 1.6 and 4.8 kyr) oscillations that likely reflected changes in redox conditions during the mid-Cenomanian^21^. A marine sediment record of early Campanian age from the South Atlantic also shows ~ 7 kyr periodicities^33^, which correspond to the duration of Pleistocene Heinrich events^62^. These millennial-scale periodicities observed in the marine sedimentary records are in good agreement with the periodicities detected in our lake record (Table 1), suggesting a potential link between the continental climate and ocean dynamics on millennial timescales.

Modeling of the Cretaceous climate–ocean system also reproduces shifts of deep-water formation sites between the southern and northern hemispheres, resulting in bi-polar seesaw-type oscillations with an Aptian–Albian palaeogeographic setting and increased *p*CO_2_^30^. Another simulation of the mid-Cretaceous climate–ocean system further indicates multiple steady states in centennial- to millennial-scale climatic variability with weaker/stronger meridional overturning circulation in the North Pacific under high *p*CO_2_ conditions^31^. Therefore, we propose that the millennial-scale extreme drought events observed in mid-latitude palaeo-Asia were possibly linked to oscillations in the strength of deep-water formation under warmer ‘greenhouse’ conditions, in conjunction with a specific palaeogeographic setting during the late Early Cretaceous.

In summary, our findings point to the existence of orbitally-induced millennial-scale climatic oscillations occurring in the late Early Cretaceous ‘greenhouse’. External solar activity forcing was effective on decadal–centennial timescales, but the millennial-scale variations were possibly amplified by internal processes, such as oscillations in the strength of deep-water formation. Together with recent report of DO-like oscillations during the Jurassic^22^, and other records of the ‘greenhouse’ intervals (i.e., Eocene^19^, Triassic^60^, Devonian^61^; Table 1), millennial-scale climatic oscillations could have existed even under warmer ‘greenhouse’ conditions. Furthermore, some studies have pointed out that variations in continental hydrologic cycle led to changes in sea level through the groundwater driven eustasy during ‘greenhouse’ period^72–74^. Although the evidence for millennial-scale sea-level changes in the ‘greenhouse’ period has not yet been presented, it is possible that this process also contributed to the co-modulation of terrestrial climate and oceanic dynamics during the Cretaceous. To test this hypothesis and for a better understanding of the mechanisms causing millennial-scale variability, further studies examining the relationship between different types of climatic state, land–ocean distribution and millennial-scale variability from other geological records are required.

It is noteworthy that recent studies suggest the possibility of multi-millennial-scale climatic instability caused by the oscillations in the strength of North Atlantic deep-water ventilation during the warmer ‘interglacial’ periods such as MIS 5e, 11, and 19^75–77^. We also note that modeling of a global warming climate–ocean scenario (4 × *p*CO_2_) produced DO-like millennial-scale oscillations in the strength of deep-water formation in the Southern Ocean^78^. Another modeling study suggests a reduction in the strength of the Atlantic meridional overturning circulation (AMOC), and switching of deep-water formation sites from the North Atlantic to the Southern Ocean, in 4 × *p*CO_2_ scenario^79^. Ongoing weakening of the AMOC^80^ may indicate that a shift towards an irreversible warmer climate state has already begun and climate tipping points are approaching^23,81^. While it is premature to speculate that the millennial-scale extreme drought events in the Cretaceous is analogous to today’s increase of extreme weather events, our data provide the geological evidence that the global climate system will potentially shift towards more unstable conditions on millennial timescales with continued increase in atmospheric CO_2_ concentrations.

## Methods

### Mongolian lacustrine deposits and the CSH01 core

Annually laminated lacustrine deposits (Shinekhudag Formation) are widely distributed in the southeastern part of Mongolia (Fig. S1). In order to obtain a continuous succession of non-weathered sedimentary strata, two scientific research cores (CSH01, CSH02) were drilled at the type section (Shine Khudag locality) in 2013 and 2014^39^. The CSH02 core (192 m core length) covers the interval from the middle Shinekhudag Formation up to the lower part of the overlying Khukhteeg Formation (Figs. S2–S4). The CSH01 core (150 m core length) covers the lower to upper Shinekhudag Formation (Figs. S5, S6). The upper part of the CSH01 core (Box 4–1 to Box 10–1; 29.6 m length) was mainly used especially for varve lamina analysis in the present study. Based on radiometric chronology by U–Pb dating of zircon by LA-ICPMS (Laser Ablation Inductively Coupled Plasma Mass Spectrometry) of intercalated tuffs above and below the formation, the depositional age of the Shinekhudag Formation is assigned to late Barremian–early Aptian ranging between 123.8 ± 2.0 and 119.7 ± 1.6 Ma (Fig. S1D)^39^. The calculated sedimentation rate of the formation ranges between 4.7 ± 2.6 and 10.0 ± 7.6 cm/kyr.

### XRF core scanner analysis and orbital-scale periodicities

The strata consist mainly of rhythmical alternations of dark grey shale, grey dolomitic marl and light grey dolomite, interpreted to reflect lake-level changes^39^. In order to obtain semi-quantitative changes in elemental composition, we performed XRF core scanner analyses (Cox, Itrax) at 500 μm spacing (~ 6 year resolution) for a 60.6 m-long section of the CSH02 core (Fig. S2A) and a 29.6 m-long section of the CSH01 core (Fig. S5A). XRF core scanner analysis was carried out at the Center for Advanced Marine Core Research, Kochi University, with a Mo tube operated at 30 kV and 55 mA and an exposure time of 10 s. Obtained Ca/Ti ratios in both the CSH01 and CSH02 cores show a marked correspondence with changes in lithology, and modulated by decimeter- to meter-scale cyclicity.

MTM spectrum of log (Ca/Ti) ratio in CSH02 core shows dominant spectral peaks of ~ 19 cm, 29 cm, 48 cm, 57 cm, 79 cm, 92 cm, 1.58 m, 2.09 m, 2.63 m, 4.33 m and 12.12 m (Fig. S3D). MTM spectrum of log (Ca/Ti) ratio in CSH01 core shows dominant spectral peaks of ~ 12 cm, 18 cm, 21 cm, 26 cm, 38 cm, 83 cm, 1.36 m, 1.97 m, 3.69 m and 9.85 m (Fig. S6D). The measurement of varve lamina thickness indicates that the average thickness of the organic–calcite varve^43–45^ laminae in shale layers is about 40–80 μm, in contrast to about 100–160 μm in evaporitic-type^41,42,82^ varve laminae partly preserved in dolomitic marl layers (Fig. S7). Based on this varve thickness, mean sedimentation rate is estimated as ~ 6–13 cm/kyr, basically agreeing with calculated values based on chronological constrains (between 4.7 ± 2.6 cm/kyr and 10.0 ± 7.6 cm/kyr)^39^. In this case, spectral peaks of the ~ 2.09 m, 2.63 m, 4.33 m, and 12.12 m of the CSH02 core correspond to 16.1–34.8 kyr, 20.2–43.8 kyr, 33.3–72.2 kyr, and 93.2–202.0 kyr, which are in good agreement with ~ 20 kyr precession, ~ 40 kyr obliquity, and ~ 100 kyr short eccentricity cycles, respectively (Fig. S3D). In the case of CSH01 core, spectral peaks of the ~ 1.97 m, 3.69 m, and 9.85 m correspond to 15.2–33.8 kyr, 28.4–61.5 kyr, and 75.8–164.2 kyr, which are also in good agreement with precession, obliquity, and short eccentricity cycles (Fig. S6D). Therefore, orbital-scale hydrological variations and resulted lake-level changes are interpreted as first-order control of lithologic changes in the Shinekhudag Formation.

### Correlation coefficient (COCO) and evolutionary fast fourier transform (FFT) analysis

Automated frequency rate method using the COCO technique^83,84^ provides statistical assessments of mean sedimentation rates in both CSH02 and CSH01 cores as well as interpretations of cycles in terms to Milankovitch orbital forcing. The power spectrum of log (Ca/Ti) ratio in CSH02 core shows dominant spectral peaks of ~ 1.58 m, 2.09 m, 2.63 m, 4.33 m and 12.12 m (Fig. S3D). The COCO between these spectral peaks and seven astronomical frequencies (i.e., 405 kyr, 125 kyr, 95 kyr, 37 kyr, 23 kyr, 22 kyr, and 18 kyr cycles) in the target La2004 solution (Laskar et al.^85^) at 121 Ma is estimated for a range of sedimentation rates from 5 to 20 cm/ kyr with a step of 0.3 cm/kyr. Both COCO and null hypothesis results show an optimal sedimentation rate of ~ 12.0 cm/kyr (Fig. S3A). The Evolutionary FFT analysis reveals changes in sedimentation rate (~ 11–13 cm/kyr) within three sections (lower 20 m, middle 20 m, and upper 20 m sections) in CSH02 core (Fig. S3B). On the basis of these changes of sedimentation rate, original thickness of log (Ca/Ti) ratio data is converted into time domain (sedimentation rate-calibrated data; Fig. S3C). MTM spectrum for sedimentation rate-calibrated log (Ca/Ti) data yield distinct ~ 22.1 kyr, 37.2 kyr, and 106.9 kyr cycles (Fig. S3E), which are in excellent agreement with precession, obliquity, and short eccentricity cycles, respectively.

We also performed COCO and Evolutionaly FFT analysis on log (Ca/Ti) ratio of CSH01 core. Both COCO and null hypothesis results show optimal sedimentation rates of ~ 8.3–10.0 cm/kyr, with four astronomical frequencies in the target (i.e., 39 kyr, 23 kyr, 22 kyr, and 19 kyr cycles; Fig. S6A). Attributed to envisioned weaker eccentricity signal in log (Ca/Ti) ratio in CSH01 core, short and long eccentricity frequencies are excluded from original La2004 solution. Measurement of varve thickness analysis and comparison with Ca/Ti ratio (Fig. S7A) suggest ~ 8.3 cm/kyr is more likely for mean sedimentation rate in CSH01 core. The Evolutionary FFT analysis results also suggest that the sedimentation rates of log (Ca/Ti) ratio in CSH01 core modulated between ~ 7 and 9 cm/kyr (Fig. S6B). On the basis of stratigraphic changes of sedimentation rates estimated from varve thickness measurement (see next chapter; Fig. S7), original thickness data of log (Ca/Ti) ratio is converted into time domain data (Figs. S5, S6C). Although statistical significance of COCO analysis for CSH01 core is not prominent, which are probably related to the shorter studied interval than that of CSH02 core, MTM spectrum of sedimentation rate-calibrated log (Ca/Ti) data yield distinct ~ 16.6 kyr, 23.4 kyr, 43.3 kyr, and 110.9 kyr cycles (Fig. S6E), which are also in good agreement with orbital precession, obliquity, and short eccentricity cycles, respectively.

These results strongly support the interpretation that log (Ca/Ti) ratio of the Shinekhdag Formation (both CSH01 and CSH02 core) has an astronomical signal. Variations in sedimentation rate-calibrated log (Ca/Ti) ratio also show a marked AM by the 100-kyr and 400-kyr eccentricity cycles in CSH02 core (Figs. S2B, S4A), and by 22-kyr precession and 100-kyr short eccentricity cycles in CSH01 core (Fig. S5B). In addition, MTM power spectrum also show pronounced spectral peaks of millennial-scale periodicities (i.e., ~ 1.4–1.5, 1.6–1.7, 1.9–2.0, 3.7–4.2, 6.6–7.8 kyr; Figs. S3E, S6E). Wavelet and window spectral analysis of both CSH01 (Fig. 4, Fig. S12) and CSH02 cores (Fig. S4) also show similar millennial-scale periodicities. Therefore, both orbital- and millennial-scale evaporation/precipitation changes are preserved in the Shinekhudag Formation.

### Relative age model by varve thickness and evaluation of dominant periodicities using a Monte-Carlo approach

To obtain the temporal variations of the evaporation/precipitation proxy (Ca/Ti), we had to calibrate differences in sedimentation rates within the lithofacies. Measurement of varve thickness of CSH01 core indicates average thickness is ~ 40–80 μm in shale layer and ~ 100–160 μm in dolomitic marl layer. Thus, we calculate the relative age model of CSH01 core by using the general relationship between Ca/Ti values and varve thicknesses (Fig. S7A). In order to ascertain potential effects caused by uncertainties in the relationship between Ca/Ti values and varve thicknesses, we performed random Monte-Carlo calculations to evaluate the changes in the relative age models (Fig. S7C) and the resulting effects on the obtained periodicities (Fig. 4B). First, the relationship between Ca/Ti values and varve thickness in the CSH01 core was clustered using Gaussian mixture models. Second, linear regressions of both log-scaled Ca/Ti values and log-scaled varve thicknesses were applied on two clustered groups (Fig. S7B). Third, uncertainties in varve thickness estimates from given log-scaled Ca/Ti were evaluated from a total of 1000 runs of Monte-Carlo approaches (estimations were derived from randomly made coefficients from aforementioned linear regressions) using the Matlab software (Fig. S7A). Estimated varve thickness was then converted into a relative age model (time interval) at each Monte-Carlo run. Time intervals at studied core depth were finally evaluated from median values of a total of 1000 Monte-Carlo runs (Fig. S7C). Our approach of relative age-model reconstruction based on varve thickness yields much smaller error estimates (more than one order of magnitude) compared to the age constraints derived from absolute dating. The 50-year moving average of obtained log (Ca/Ti) variations (sedimentation rate calibrated data) are shown in Figs. 3F, 4A and 5A.

After the calculation of 1000 different ages using the aforementioned relative age model (Fig. S7C), then FFT Periodogram Power Specta^86^ at different 1000 ages were calculated (semitransparent grey lines in Fig. 4B), in order to ascertain the bias on spectrum-frequency domain derived by age model uncertainty. Median Power Spectrum were subsequently evaluated from 1000 different FFT spectra (thick black lines in Fig. 4B). Significances (90%, 95%, and 99% confidence level) are estimated over the median power spectrum with the assumption that the subjected power spectrum contains red-noise as its back ground signal^87^. FFT power spectra of the selected time windows (i.e., drier and wetter intervals of the 100-kyr eccentricity minimum/maximum) were calculated for data at randomly generated age, taking into account the age-depth uncertainties described above (Fig. 4B). A total of 1000 times FFT with random age were resampled at the same frequency-power domain. Calculations of Monte-Carlo approach, FFT estimation, and red-noise significance test were calculated using the Matlab software. Shape of random age FFT (semitransparent grey lines) and their Medians (thick black lines) demonstrate their maxima at ranging from few hundreds to several tens of kyr, indicatives of certain periodicities even with consideration of age uncertainties. Furthermore, there are prominent periodicities in centennial-scales, while more quasi-periodic pattern occurs in millennial-scales (Fig. 4B).

### MTM spectrum, Gaussian band-pass filtering, and amplitude modulation (AM) analysis

Potential bias derived from choice of different spectral analysis was further compared in Fig. S12 with Mont-Carlo approach FFT (left panels) and MTM (right panels). The obtained millennial-scale periodicities are overall consistent in both FFT spectral analysis of Monte-Carlo random run and MTM spectrum (Fig. S12). We also performed ‘multiple testing’ of spectral analysis by Bending power law method^88,89^ (Fig. S15). MTM spectrum and Bending power law method was calculated using the *Acycle* software^84^.

Gaussian band-pass filtering of subject data were performed using the AnalySeries software^90^. AM of subject data were obtained by the envelope analysis with the Hilbert transform using Matlab software with open script of Envelope secant method^91^. Envelopes of millennial-scale periodicities in log(Ca/Ti) high frequency data show distinct AM of the 100-kyr eccentricity cycle (Fig. 5A). In addition, envelopes of 1.5-kyr filtering of log(Ca/Ti) data show AM of the 40-kyr obliquity cycle (Fig. 5B).

### Reconstruction of algal OM flux by varve lamina analysis

Shale beds representing lake level highstand show a distinct micrometer-scale lamination (Fig. 1C, 1D, Fig. S1C). Due to the absence of diatoms in lacustrine deposits prior to the Cenozoic, identification of seasonal sublayers can be difficult. However, fluorescence microscopic inspection (OLYMPUS BX51 and DP73 with U-MNV2 mirror unit: excitation filter at 400–410 nm and absorption filter at 455 nm in Kochi University) and SEM–EDX (Hitachi SU-6600 in Nagoya University) analyses in a 5.5 cm-thick section reveals that the compositional changes observed in the deposits exhibit a clear seasonality pattern indicative of annual deposition (Fig. 1C,D). For reconstructing changes in algal productivity for a 1091-year interval (5.5 cm section), we first performed varve counting by visual investigation by using fluorescence microscopic image (Fig. S8). The organic matter-rich layers are usually laterally continuous (Figs. S1C, S8), and micritic calcite aggregates commonly appear above and/or within the organic matter-rich layers (Fig. 1C). Annual sets of lamina couplets are also confirmed by the occurrence of conchostracans within the clay layer. There are few slumping structures that cut-off the underlying lamina (Fig. S8), but prominent flood event layers^92,93^ that cut into the underlying laminae were not identified in studied 5.5 cm interval. By comparison with modern analogues of carbonate lakes^43–45^, laminae couplets are interpreted to reflect the deposition of algal OM and calcite precipitation in the lake epilimnion during late spring–summer, and clay-dominated deposition during the autumn–winter, respectively. Since coarser grains and grading structures are not observed, the lamination is considered as organic-calcite varves, different from periglacial varve. Thus, we adopt the highly fluorescent algal OM as a proxy of lake surface algal productivity.

To obtain semi-quantitative data on the temporal changes in algal OM flux, we transform the fluorescence images, that occasionally show folded patterns of algal OM layer, to one-dimensional data by using lamination tracing and unfolding program^46^ (Fig. S9). First, fluorescence images are binarized into two-dimensional RGB data, and folded patterns of highly fluorescent summer algal OM lamina are automatically extracted and stretched out. Then, two-dimensional distributions of the fluorescence intensity of stretched lamina images were converted into a one-dimensional intensity profile in a direction perpendicular to the varve lamina. A lamination tracing and unfolding program^46^ is utilized in this procedure, and a one-dimensional intensity profile is generated (Fig. S9). Finally, changes in algal OM flux are reconstructed based on the fluorescence intensity and thickness of summer algal OM micro-layers (Fig. S8). Changes in lamina thickness (both OM and clay mineral layers) are also obtained. The 3-year moving average of changes in algal OM flux, divided by varve thickness, is shown in Fig. 2A. Average thickness of laminae couplets are ca. 55 μm in the studied interval.

### Scanning X-ray analytical microscope (SXAM)

To compare elemental composition change and fluorescence intensity of algal OM in a 55 cm-thick shale dominant section of the CSH01 core, we carried out μ-XRF mapping using a scanning X-ray analytical microscope (SXAM; Horiba XGT-5000). SXAM analysis at 60 μm spacing (~ 1 year resolution) was carried out in Nagoya Municipal Industrial Research Institute with a 60 μm diameter beam of Rh radiation (50 kV, 1 mA). Two-dimensional distributions of Ca and Ti contents obtained from counting data of SXAM were then converted into a one-dimensional profile in the direction perpendicular to the layering (Fig. 3A, Fig. S10). We also showed Wavelet analysis results for both depth domain data of Fluorescence intensity and Ca count in Fig. S10.

### Wavelet analysis

To examine the periodicities of the variations in the algal OM productivity and log (Ca/Ti) ratio, the obtained data were subjected to wavelet analysis using ‘Morlet’ as mother wavelet by the Scilab software. The vertical axis of the plots represents the periodicity, while the horizontal axis corresponds to time. We also performed wavelet analysis and wavelet coherence analysis using the Matlab software. Comparison between wavelet analysis by Scilab and Matlab is also presented in Fig. S16.

### Stable isotope analysis

Measurements of stable carbon and oxygen isotopes of sedimentary carbonates were carried out on powdered bulk rock material (~ 2.5 cm spacing, ~ 0.5 mg) on a total of 183 samples (Fig. 3F). Stable isotope analysis was conducted using a Thermo Fisher Scientific Gasbench II carbonate device connected to a Thermo Fisher Scientific Delta V Advantage IRMS, available at the Leibniz University Hannover, Germany. The gas bench uses viscous water-free (98 g/mol) orthophosphoric acid at 72 °C to release CO_2_ of the calcite from the sample material 1 h before the start of the measurement. Repeated analyses of certified carbonate standards (CO-1, NBS-18, NBS-19) show an external reproducibility ± 0.08‰ for δ^18^O_carb_ and ± 0.06‰ for δ^13^C_carb_. Values are expressed in conventional delta notation relative to the Vienna-Pee Dee Formation belemnite (VPDB) international standard, in per mil (‰).

The obtained δ^18^O_carbonate_ value shows marked correspondence with log (Ca/Ti) ratio as well as lithofacies (Fig. 3F). Dark grey shale layer (low Ca/Ti ratio) show very negative values (between − 10 and − 7‰), while dolomite layer (high Ca/Ti ratio) show less negative values (between − 4 and − 2‰). The large isotopic variations (> 8‰) between dolomite and shale layers support a scenario of drastic changes in evaporation/precipitation. The observed magnitude of the isotopic variations are in accordance with the reported variations in δ^18^O_carbonate_ value from the mid-latitude lake sediment records covering the last glacial and deglaciation interval^56,94–96^.

### Lacustrine varve types and relationship with climate regimes

In order to verify the significance of the millennial-scale evaporation/precipitation changes recorded in the Shinekhudag Formation, we compiled a Holocene lacustrine varve record (modified after previous studies^36,37^ and additional references listed in Table S1). Figure S11 shows the global distribution of different types of lacustrine varves and sediments, such as organic-clastic varves, organic-calcite/siderite varves, evaporitic varves, and dolomitic deposits. Organic-clastic varves and organic-calcite varves predominantly form in Holocene deep lakes located in mid-latitude humid climates^43–45^. On the other, lacustrine primary dolomite mostly forms in shallow saline lakes under subtropical semi-arid climatic conditions^41,42,82^. Thus, the periodic occurrence of dolomite beds in the Cretaceous lake record likely reflects millennial-scale drought events and drastic switches in climatic conditions.

### Comparison between Cretaceous lake record and Antarctic ice-core record

Based on the thermal bipolar seesaw model, Greenland temperature variability record for the last 800 kyr (GL_T__syn) were reconstructed from δ^18^O of Antarctic ice core record (EDC)^7^. We compared the climatic variability of both our Cretaceous evaporation/precipitation variation (~ 355 kyrs interval) and the last 400 kyr interval of Greenland temperature record (GL_T__syn_high) (Fig. 5, Figs. S13, S14). Wavelet analysis and wavelet coherency analysis reveal that nearly similar periodicities of millennial-scale oscillations (i.e., ~ 1.0, 2.0, 3.6, 5.3, and 7.2-kyr periodicities) were evident in both records (Fig. S13).

## Supplementary Information

Supplementary Information.

Supplementary Table S1.

Supplementary Table S2.
