# Supplementary material for: Decadal–centennial-scale solar-linked climate variations and millennial-scale internal oscillations during the Early Cretaceous
Source: Sci Rep. 2022 Dec 19;12:21894. doi: 10.1038/s41598-022-25815-w (PMC9763356; doi:10.1038/s41598-022-25815-w)
Supplement: Supplementary file 1 — Supplementary Information. [file 41598_2022_25815_MOESM1_ESM.pdf]

Supplementary Materials for

Decadal–centennial-scale solar-linked climate variations and millennial-scale internal oscillations during the Early Cretaceous

Hitoshi Hasegawa*, Nagayoshi Katsuta, Yasushi Muraki, Ulrich Heimhofer, Niiden Ichinnorov, Hirofumi Asahi, Hisao Ando, Koshi Yamamoto, Masafumi Murayama, Tohru Ohta, Masanobu Yamamoto, Masayuki Ikeda, Kohki Ishikawa, Ryusei Kuma, Takashi Hasegawa, Noriko Hasebe, Shoji Nishimoto, Koichi Yamaguchi, Fumio Abe, Ryuji Tada, Takeshi Nakagawa

*To whom correspondence should be addressed. E-mail: hito_hase@kochi-u.ac.jp

This PDF file includes:

Figs. S1 to S16
Table S1.
Supplementary References

The datasets presented in this study are shown in **Table S2** and **S3** as separated files.

Table S2. The 3-year moving average of Algal OM flux/Varve thickness

Table S3. The 50-year moving average of log(Ca/Ti), log(Ca/Ti) high frequency, and amplitude modulation envelopes of log(Ca/Ti) high frequency in CSH01 core

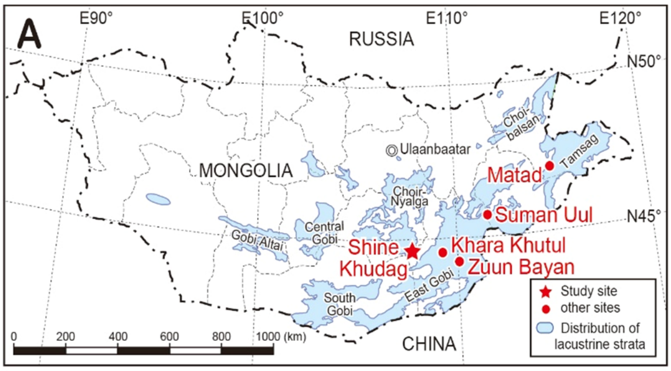

Fig. S1. **(A)** Location of study sites and distribution of lacustrine deposits in southeastern Mongolia[40]. **(B)** Outcrop photograph of shale-dominant section in the Shine Khudag locality. **(C)** Transmitted-light (left) and fluorescence (right) photomicrographs of a 6 mm thick (~110 years) interval of well-laminated shale. Brown colored laminae under transmitted light (bright green color under fluorescence) correspond to spring–summer algal OM rich layers. **(D)** Litho- and chronostratigraphic correlation of the Tsagantsav, Shinekhudag, and Khukhteeg Fms in southeastern Mongolia and obtained radiometoric age of intercalated basalt and tuffs[39]. Stratigraphic levels of **figs. S2A** & **S5A** are also shown.

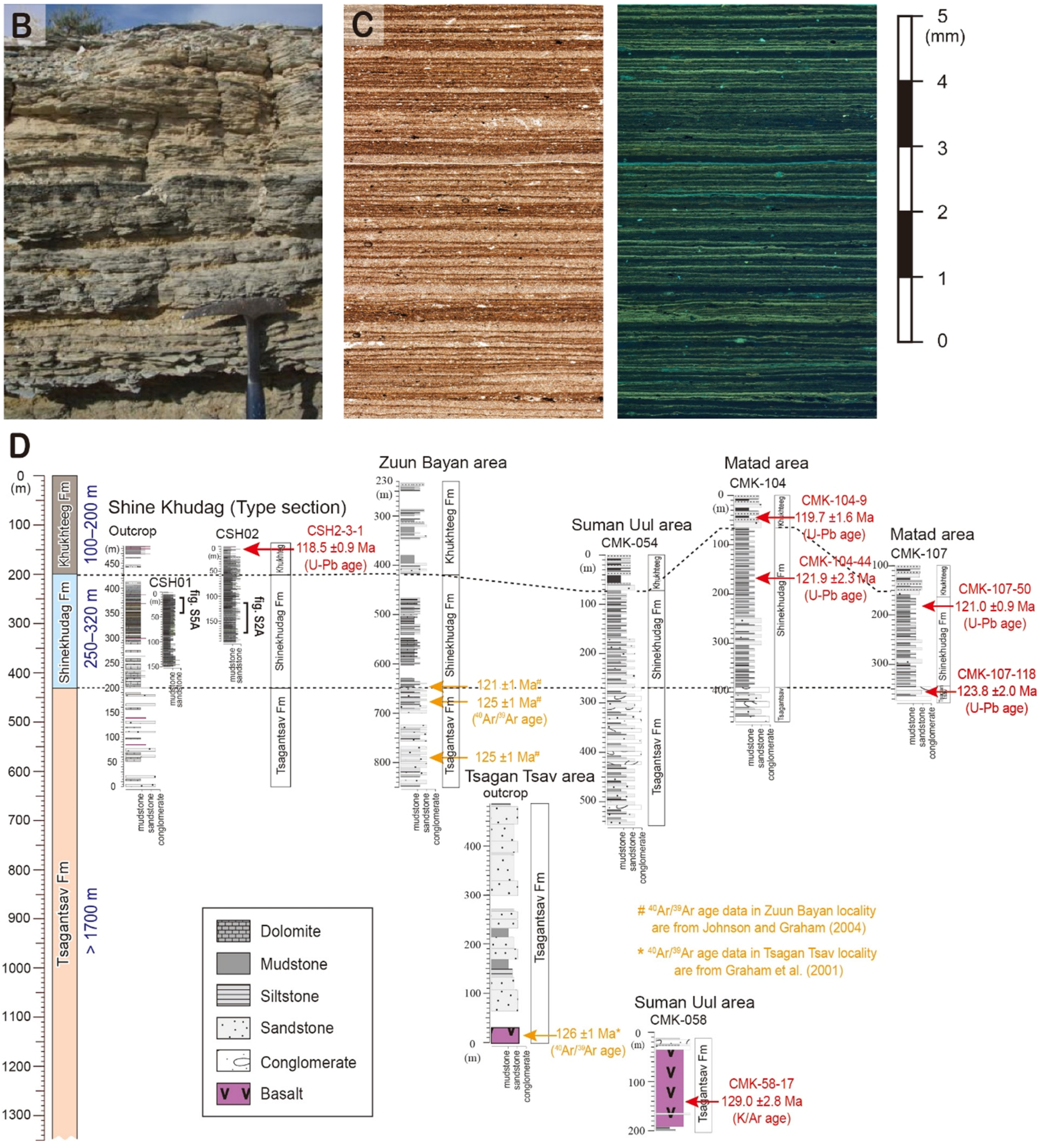

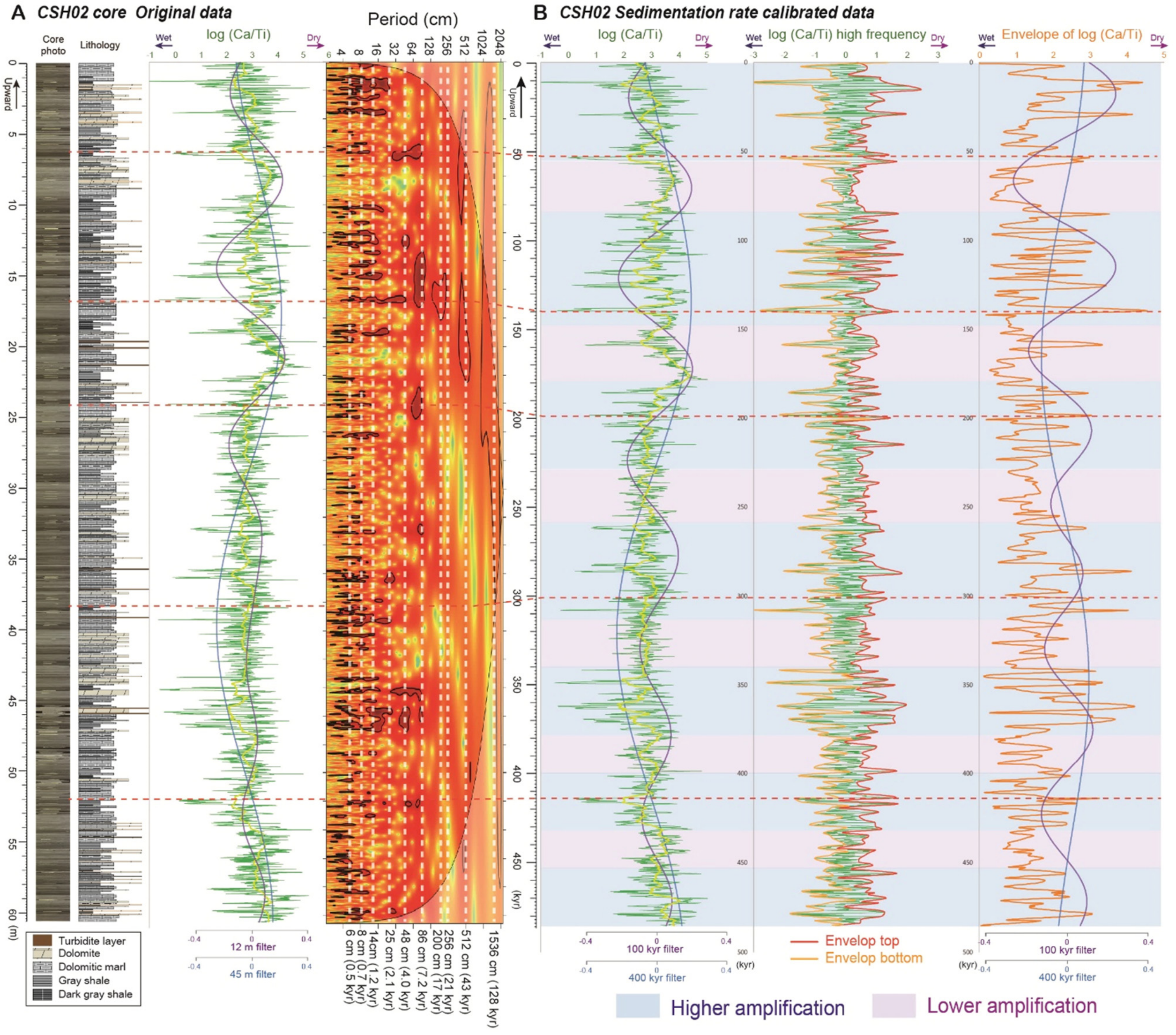

Fig. S2. Comparison between core photograph, lithologic column and original Ca/Ti ratios of a 60.6-m thick interval of the CSH02 core with Wavelet analysis results (**A**) and sedimentation rate-calibrated data of Ca/Ti ratio (~485-kyr interval) (**B**). Amplitude modulation of 12 m and 100 kyr filters (purple), 45 m and 400 kyr filters (blue), and envelop (subtraction of envelop top and bottom) of log (Ca/Ti) are also shown. Note that abrupt millennial-scale oscillations in CSH02 core show distinct amplitude modulation of both short and long eccentricity cycles.

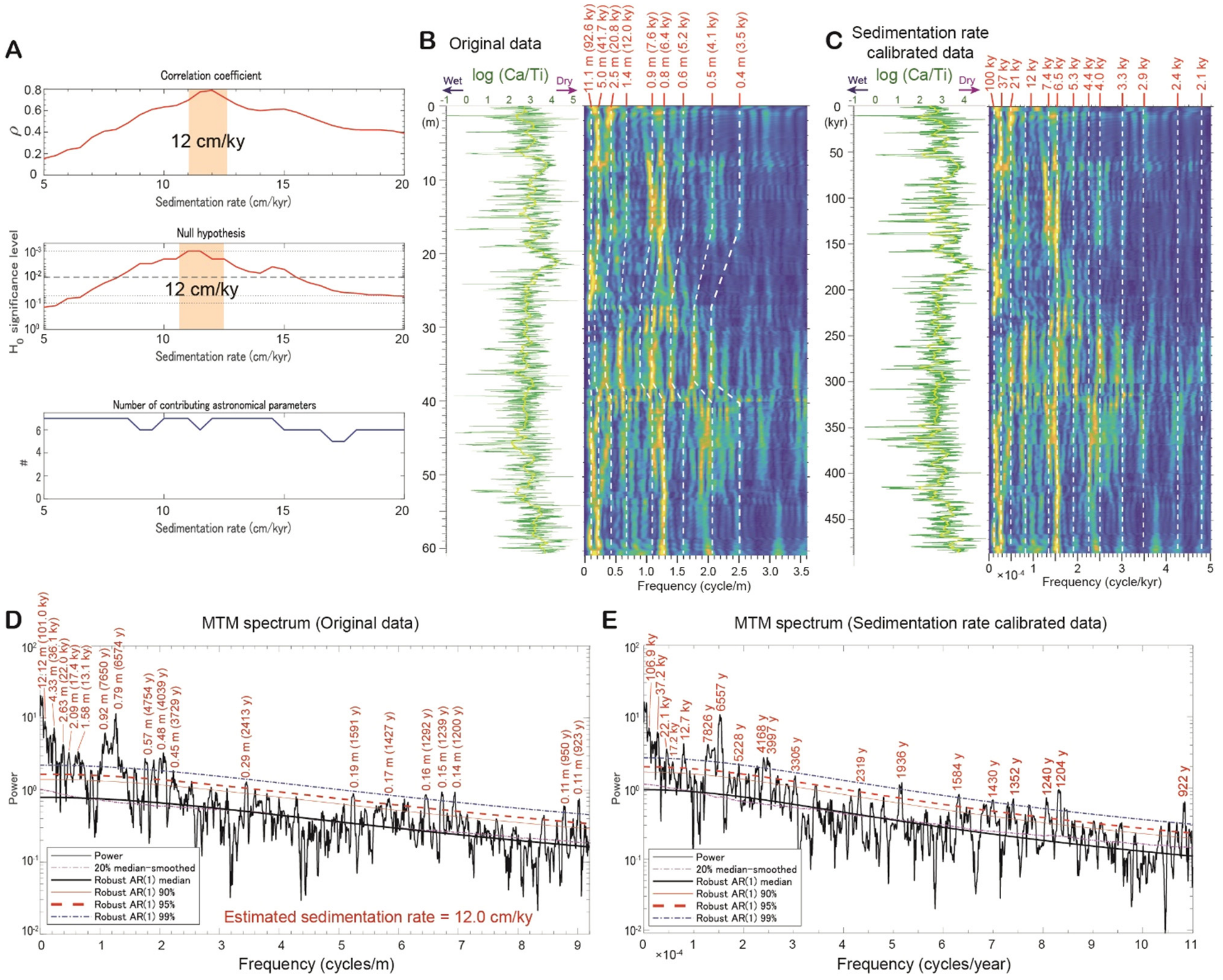

Fig. S3. Correlation coefficient (COCO) analysis **(A)**, Evolutionary power spectral analysis **(B, C)**, and Multi-taper method (MTM) power spectrum **(D, E)** of original log (Ca/Ti) data and sedimentation rate-calibrated data of CSH02 core using *Acycle* software (Li *et al.*, 2019[84]). **(A)** Both correlation coefficient and Null hypothesis results show the optimal sedimentation rates of ~12.0 cm/kyr (yellow box). The tested sedimentation rates range from 5 to 20 cm/kyr with a step of 0.3 cm/kyr, and the number of Monte Carlo simulations is 2000. The middle age of 121 Ma and all seven astronomical frequencies of Laskar04 solution were used. **(B, C)** Evolutionary power spectra of original (12 m sliding window with 0.1 m step) and sedimentation rate-calibrated data (97 kyr sliding window with 776 yr step). **(D, E)** 2π MTM power spectrum of the original log (Ca/Ti) data and sedimentation rate-calibrated data. The spectrum showing red-noise fit to the spectrum based on the conventional AR(1) model. The 20% median-smoothed spectrum is also shown (dashed pink). The red-noise fit to the spectrum based on the robust AR(1) model (thick black) and 90% (solid red), 95% (dashed red), 99% (blue dash-dot), confidence limits are based on the best fit to the log power of the median smoothed background spectrum.

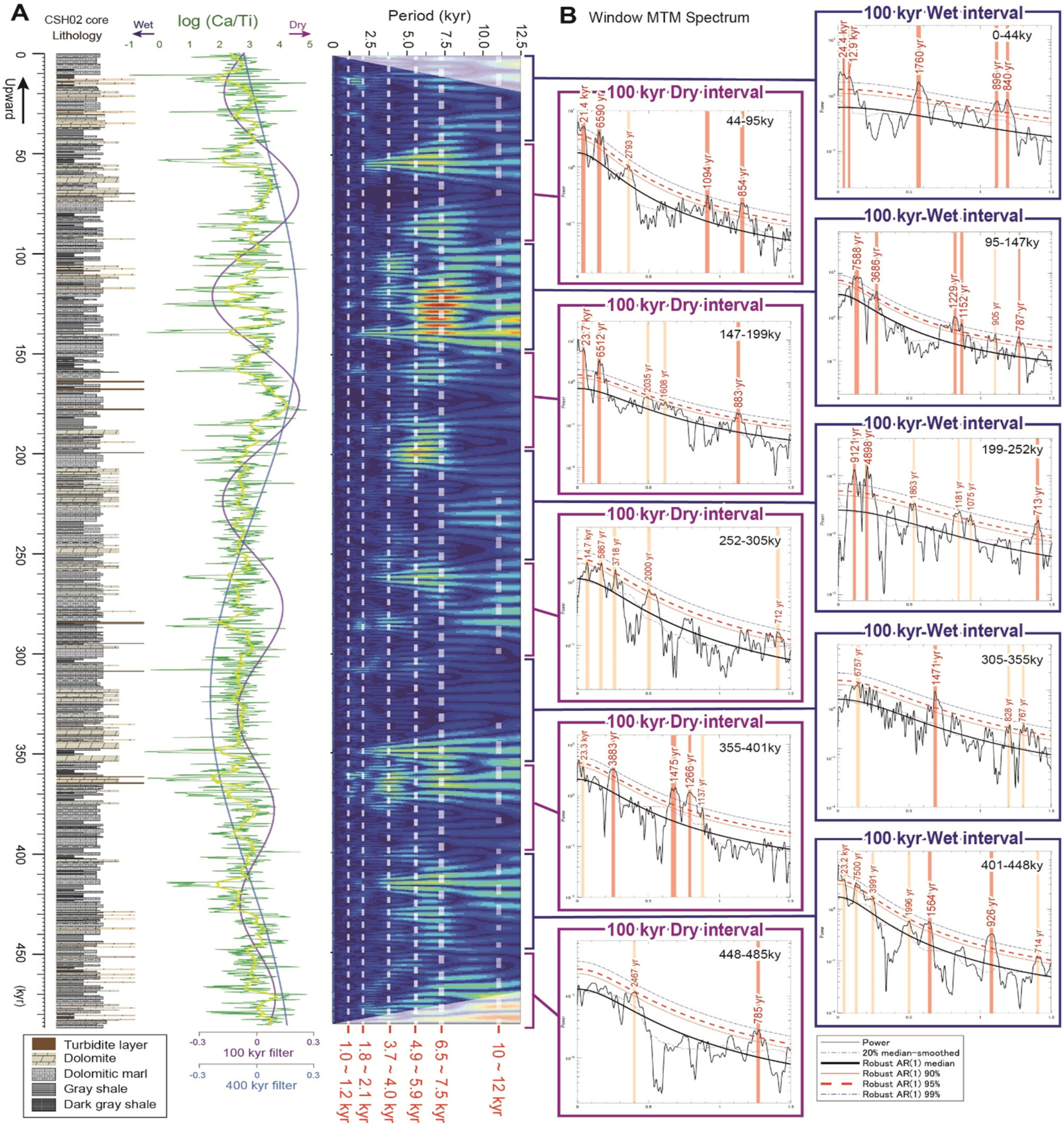

Fig. S4. Decadally resolved changes in log (Ca/Ti) data in CSH02 core for a ~485-kyr interval. **(A)** Lithology, Ca/Ti ratios and wavelet power spectrum for a 60.6 m-thick (~485-kyr) interval of core CSH02 at 500 μm (~6 year) resolution using a XRF core scanner (Cox, Itrax). Amplitude modulation of 100-kyr (purple) and 400-kyr (blue) filters are also shown. (**B**) Window MTM spectral analyses of log (Ca/Ti) variations for the drier and wetter interval of 100-kyr eccentricity minimum/maximum.

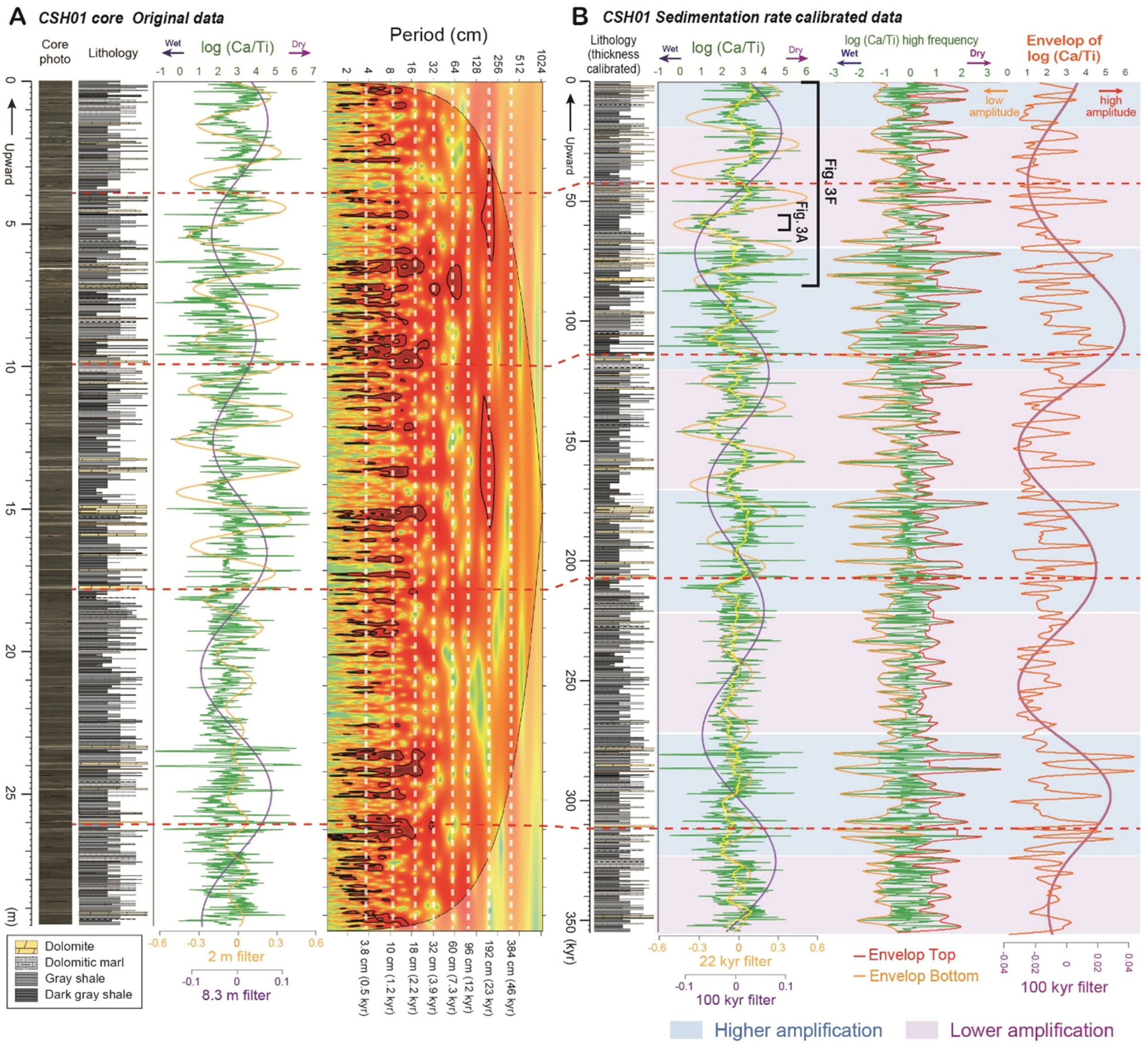

Fig. S5. Comparison between core photograph, lithologic column and original Ca/Ti ratios of a 29.6-m thick interval of the CSH01 core with Wavelet analysis results (**A**) and sedimentation rate-calibrated data of Ca/Ti ratio (~355-kyr interval) (**B**). Sedimentation rate-calibrated Ca/Ti data are used in **Figs. 3, 4, 5**. Amplitude modulation of 2.0 m and 22 kyr filters (yellow), 8.3 m and 100 kyr filters (purple), and envelop of log (Ca/Ti) are also shown. Note that abrupt millennial-scale oscillations in CSH01 core also show distinct amplitude modulation of 100 kyr eccentricity cycles.

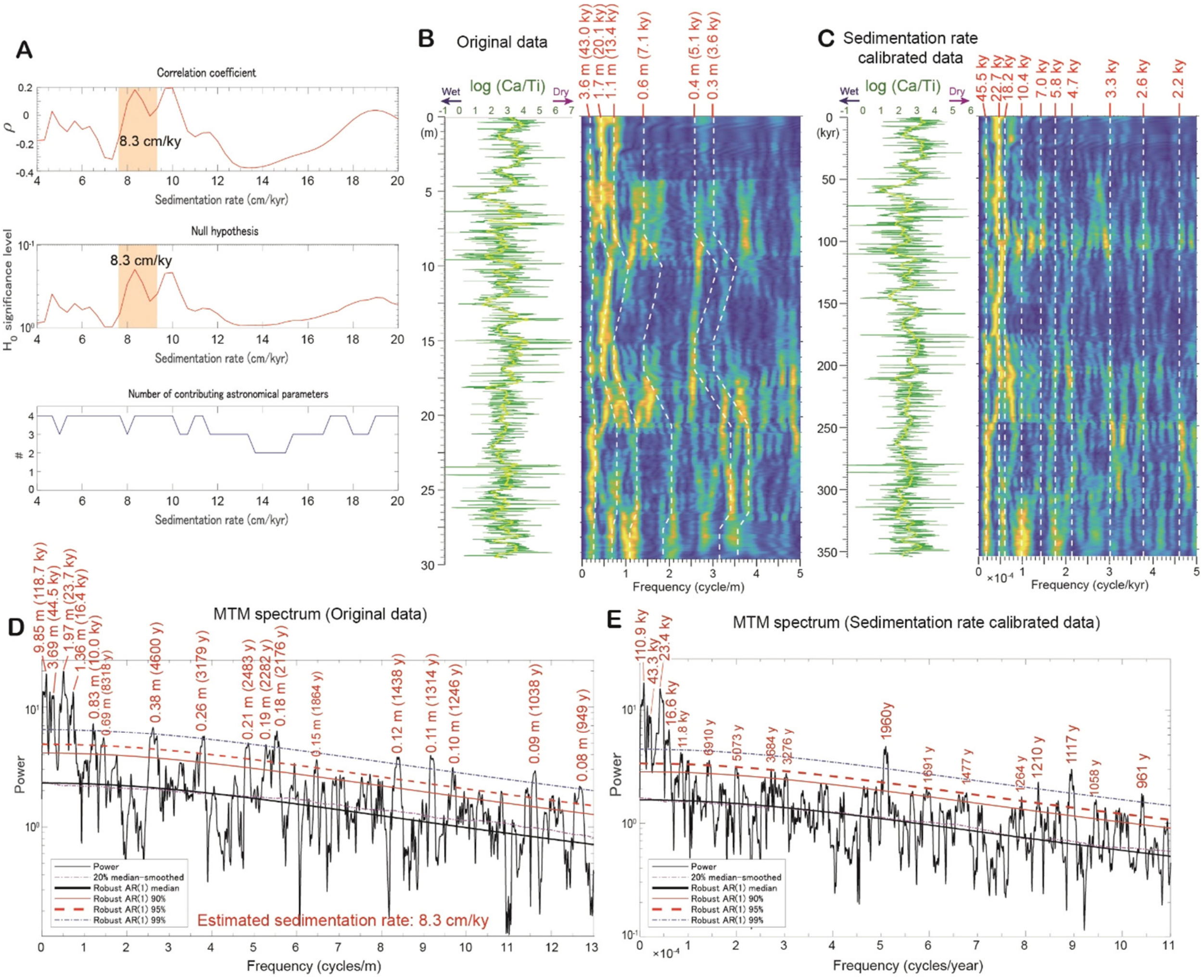

Fig. S6. COCO analysis **(A)**, Evolutionary power spectral analysis **(B, C)**, and MTM power spectrum **(D, E)** of original log (Ca/Ti) data and sedimentation rate-calibrated data of CSH01 core using *Acycle* software[84]. **(A)** Both correlation coefficient and Null hypothesis results show the optimal sedimentation rates of ~8.3 cm/kyr (yellow box). The tested sedimentation rates range from 4 to 20 cm/kyr with a step of 0.3 cm/kyr, and the number of Monte Carlo simulations is 2000. The middle age of 121 Ma and four astronomical frequencies of Laskar04 solution were used. **(B, C)** Evolutionary power spectra of original (6 m sliding window with 0.05 m step) and sedimentation rate-calibrated data (71 kyr sliding window with 568 yr step). **(D, E)** 2π MTM power spectrum of the original log (Ca/Ti) data and sedimentation rate-calibrated data. The spectrum showing red-noise fit to the spectrum based on the conventional AR(1) model. The 20% median-smoothed spectrum is also shown (dashed pink). The red-noise fit to the spectrum based on the robust AR(1) model (thick black) and 90% (solid red), 95% (dashed red), 99% (blue dash-dot), confidence limits are based on the best fit to the log power of the median smoothed background spectrum.

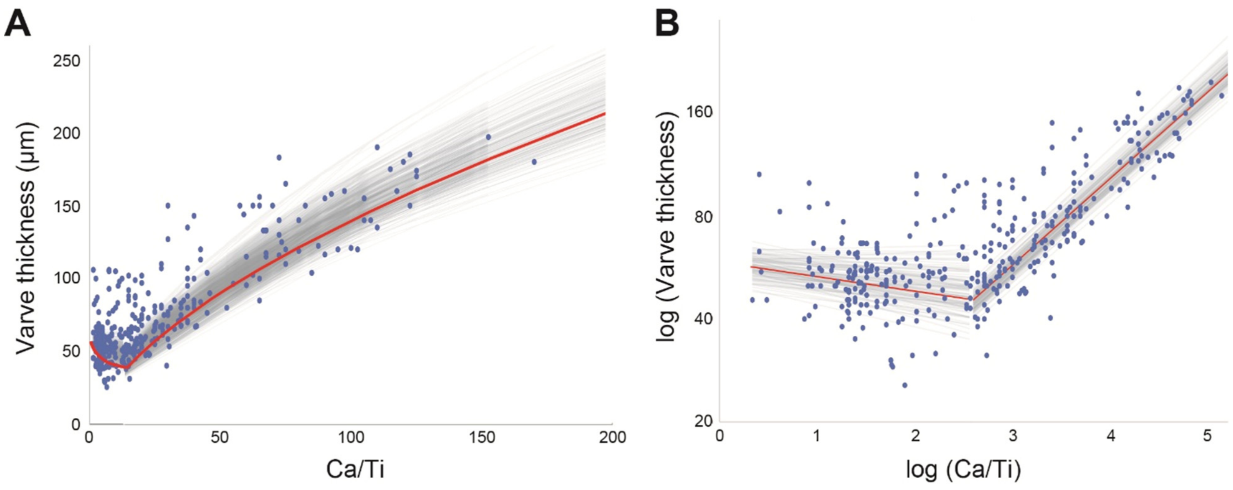

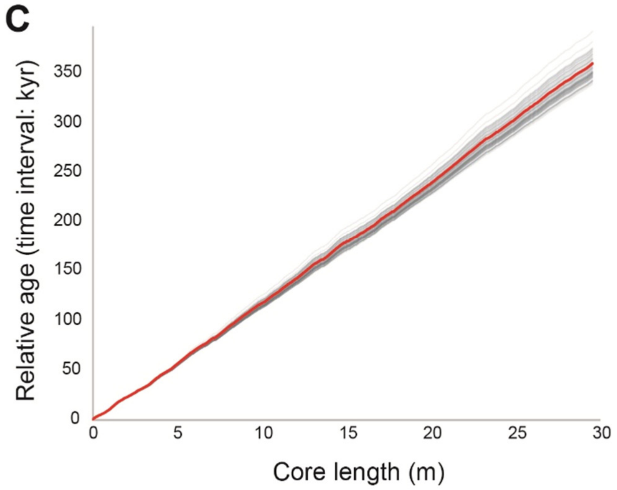

Fig. S7. Relationship between Ca/Ti and varve thickness of CSH01 core **(A)**. Probabilistic estimation of regressions from Monte-Carlo 1000 random runs are shown as gray line and their median red line. (1) logarithms of both Ca/Ti value and varve thickness were clustered into two groups using Gaussian mixture model. (2) linear regressions of both log-scaled Ca/Ti and varve thickness were applied on each given cluster **(B)**. (3) uncertainties in varve thickness estimations from given log-scaled Ca/Ti were evaluated from Monte-Carlo 1000 random runs. **(C)** Relationship between original CSH01 core length and calculated relative age (time interval: kyrs) from regressions of Monte-Carlo random runs. Time intervals at subject core length was evaluated from median values (red line) of 1000 runs. Obtained time-series of Ca/Ti variations were used for Fourier spectral analysis concerning uncertainties of relative age estimations (**Fig. 4B**).

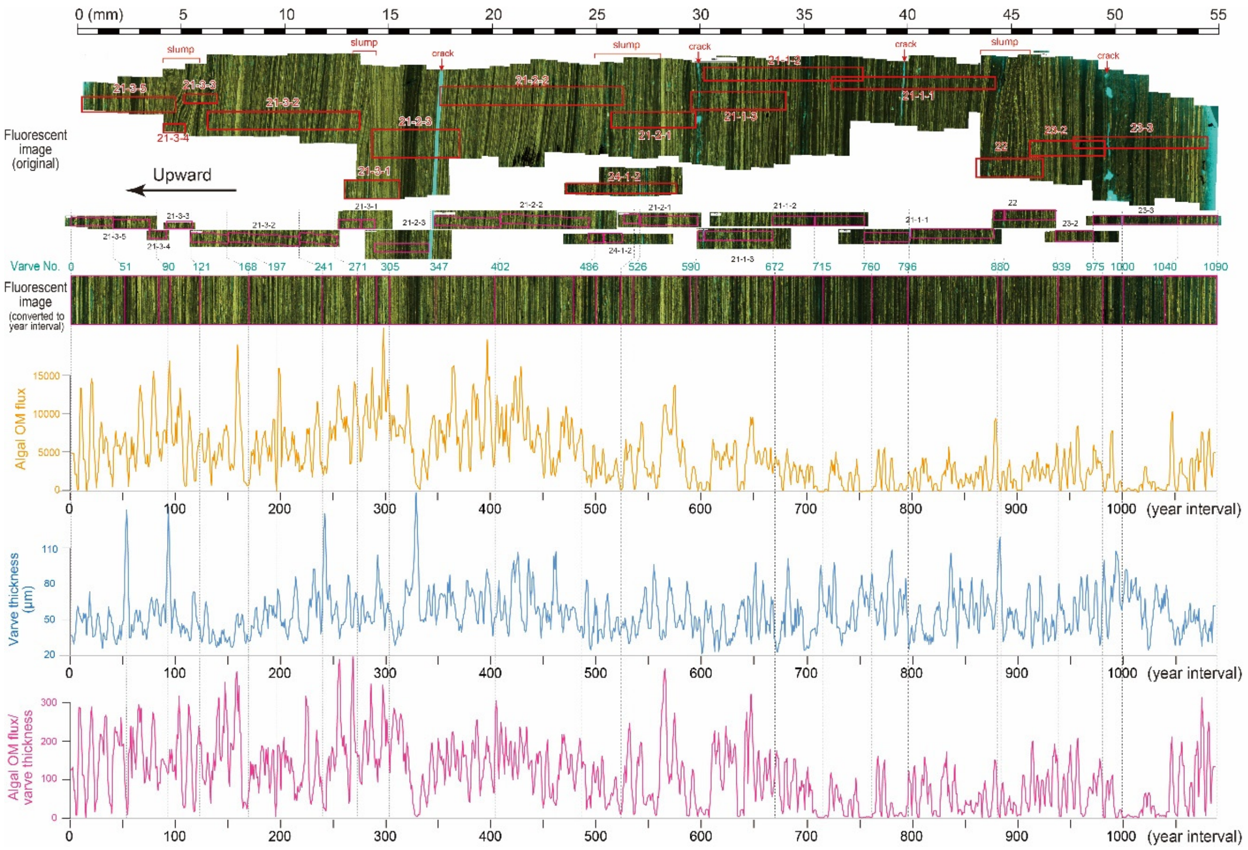

Fig. S8. Fluorescence photomicrograph of a 5.5 cm thick interval (corresponding to 1091 years) of well-laminated shale. Reconstructed changes of algal OM flux (yellow), varve thickness (blue), and algal OM flux/varve thickness (purple), are also shown.

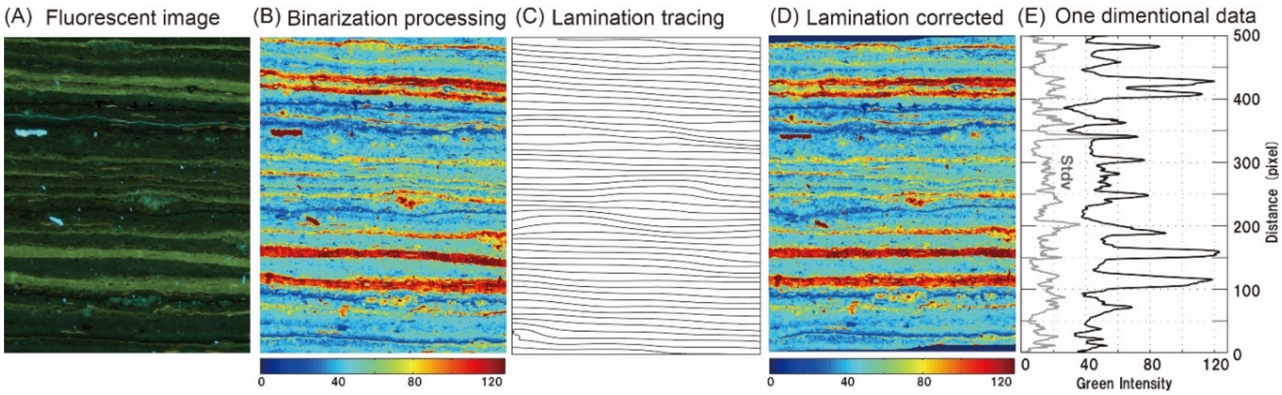

Fig. S9. Workflow image illustrating the lamination tracer and unfolding program[46], which was developed to convert the original "folded" lamination pattern into a straight "unfolded" lamination, utilized in this study. Two-dimensional profiles of fluorescence photomicrograph images are converted into one-dimensional fluorescence image, by stretching out the original "folded" lamination.

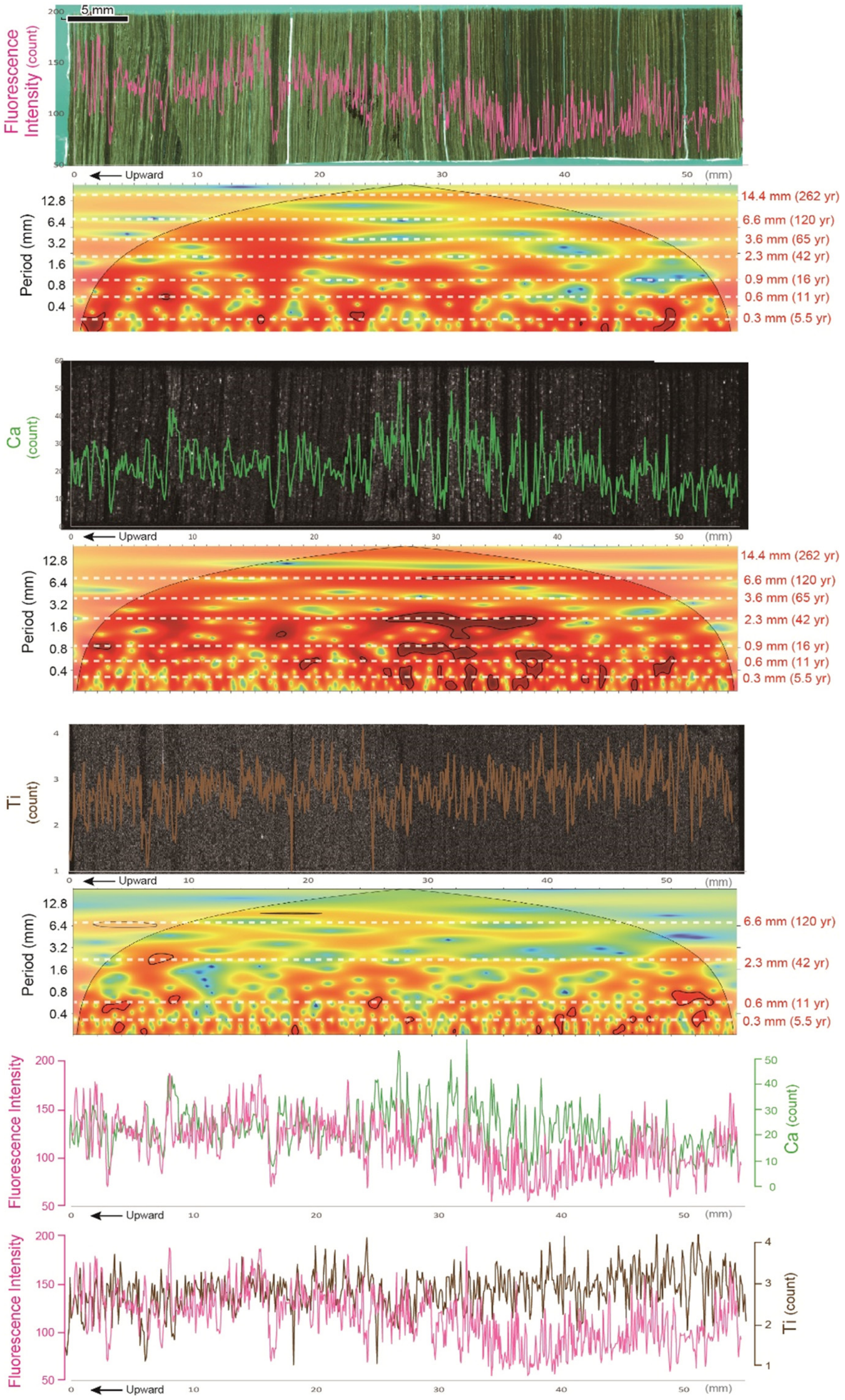

Fig. S10. Comparison between Fluorescence intensity of algal OM and obtained one-dimensional Ca and Ti profiles from SXAM analysis in a dark grey shale layer (5.5 cm interval). Note that fluorescence intensity (algal productivity proxy) are negatively correlated with Ti concentration (terreginus clay input proxy). Wavelet analysis of these data are also shown. Obtained year cycles were calculated based on the average varve thickness (i.e., 55 µm in this interval).

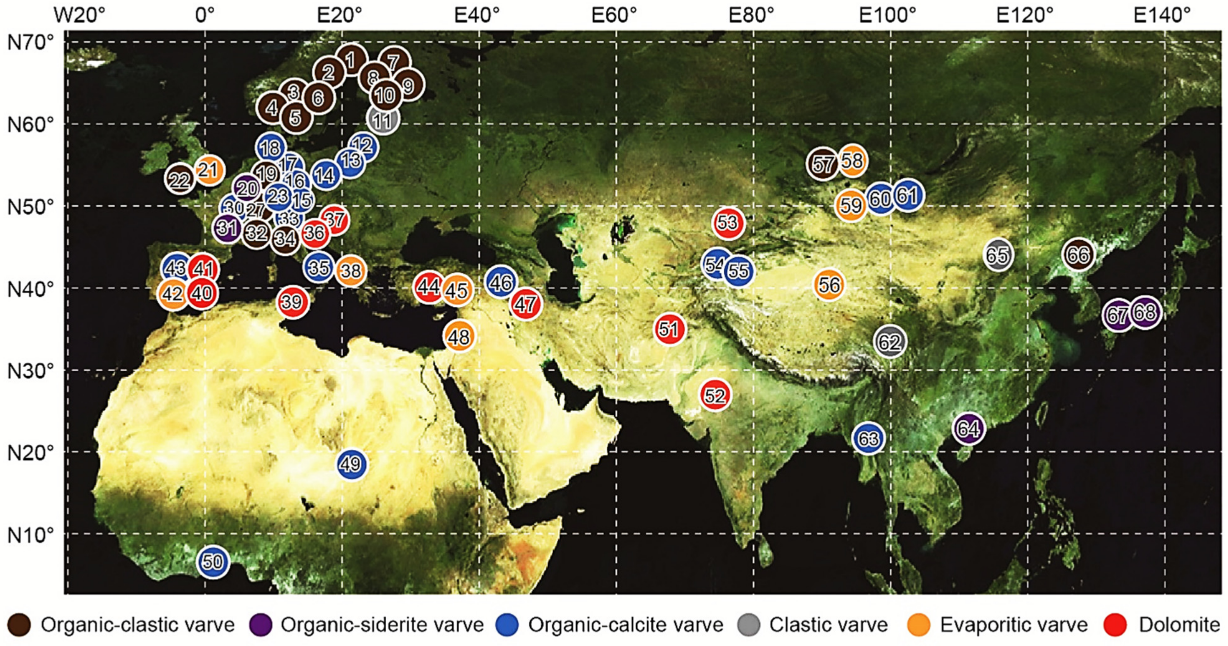

Fig. S11. Global distribution of different types of lacustrine varves and sediment, such as organic-clastic varves, organic-calcite/siderite varves, evaporitic varves, and dolomitic deposits (modified after, ref.[36,37] and additional references listed in **Table S1**).

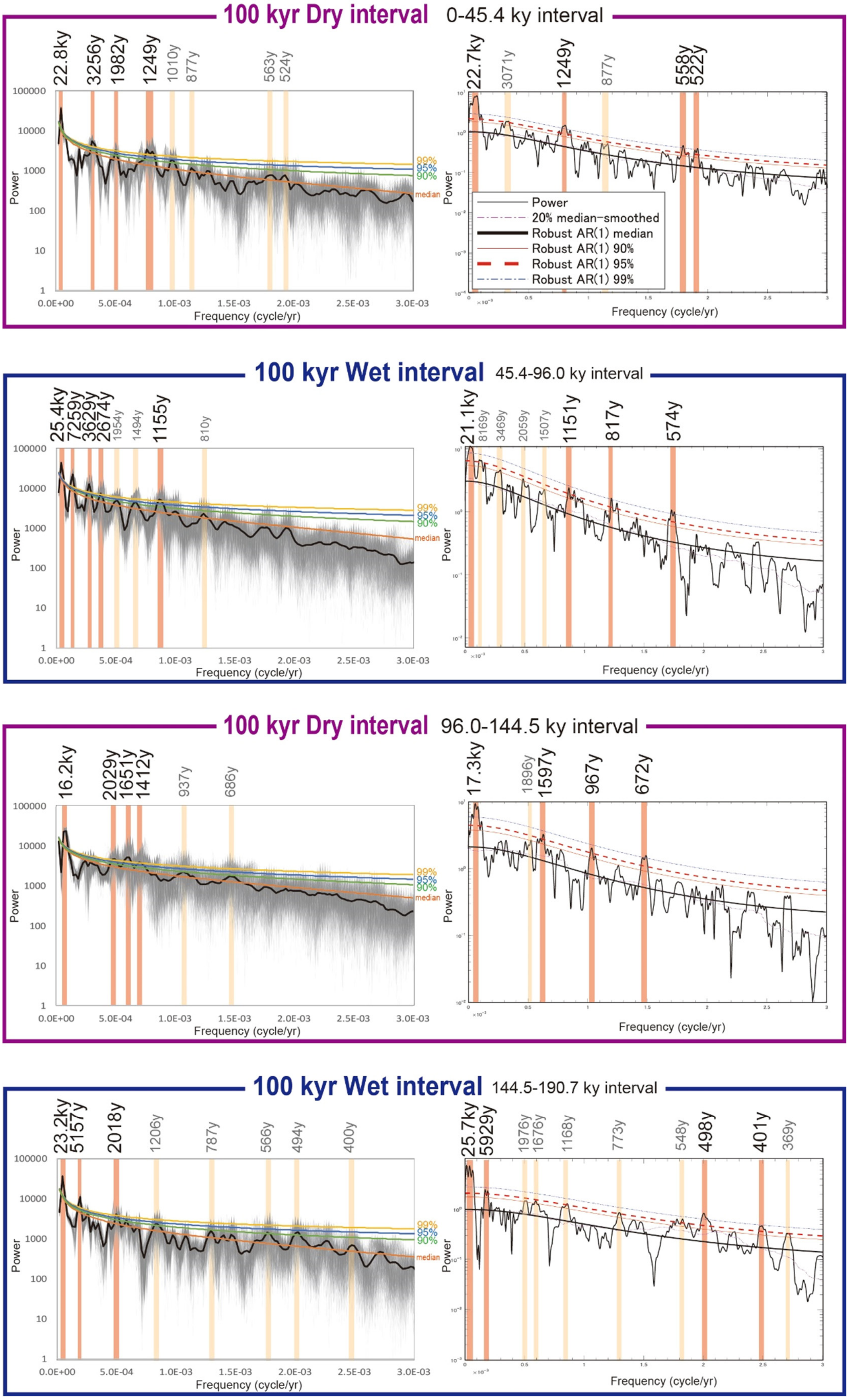
100 kyr Dry interval
0-45.4 ky interval
22.8ky
3256y
1982y
1249y
1010y
877y
563y
524y
22.7ky
3071y
1249y
877y
558y
522y
Power
20% median-smoothed
Robust AR(1) median
Robust AR(1) 90%
Robust AR(1) 95%
Robust AR(1) 99%
99%
95%
90%
median
Frequency (cycle/yr)
100 kyr Wet interval
45.4-96.0 ky interval
25.4ky
7259y
3629y
2674y
1954y
1494y
1155y
810y
21.1ky
8169y
3469y
2059y
1507y
1151y
817y
574y
100 kyr Dry interval
96.0-144.5 ky interval
16.2ky
2029y
1651y
1412y
937y
686y
17.3ky
1896y
1597y
967y
672y
100 kyr Wet interval
144.5-190.7 ky interval
23.2ky
5157y
2018y
1206y
787y
566y
494y
400y
25.7ky
5929y
1976y
1676y
1168y
773y
548y
498y
401y
369y

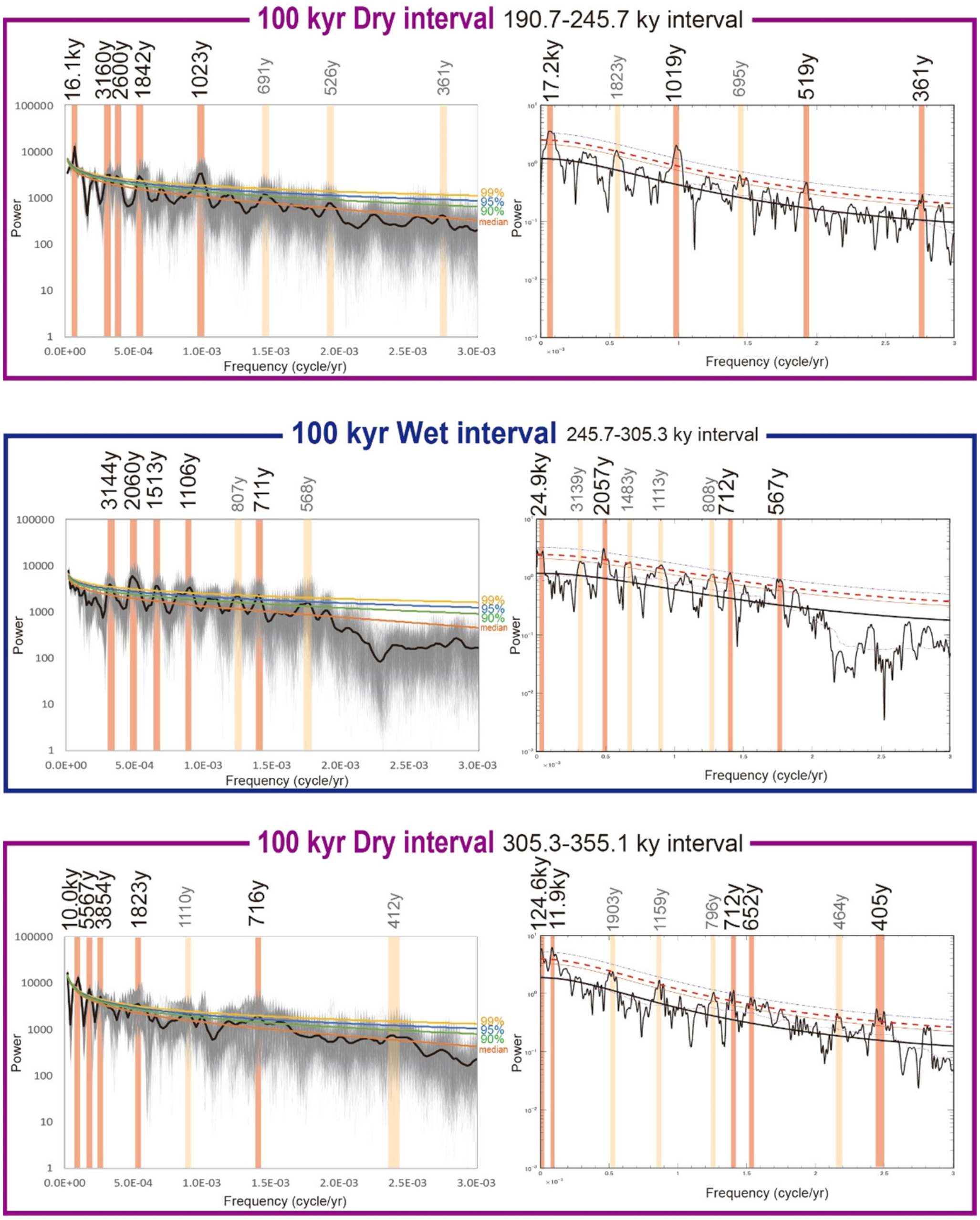

Fig. S12. Comparison of window Fourier spectral analyses of log (Ca/Ti) variations for the drier interval and wetter interval of 100-kyr eccentricity minimum/maximum. Note that both the FFT spectrum of median values of Monte Carlo random runs concerning uncertainties of relative age estimations (shown in **Fig. 4B**) and the MTM spectrum show rather consistent spectral peaks. Reddish bar and numbers indicate significant spectral peak above 99 % confidential level (CL). Yellowish bar and numbers indicate moderate spectral peak above 90 % CL.

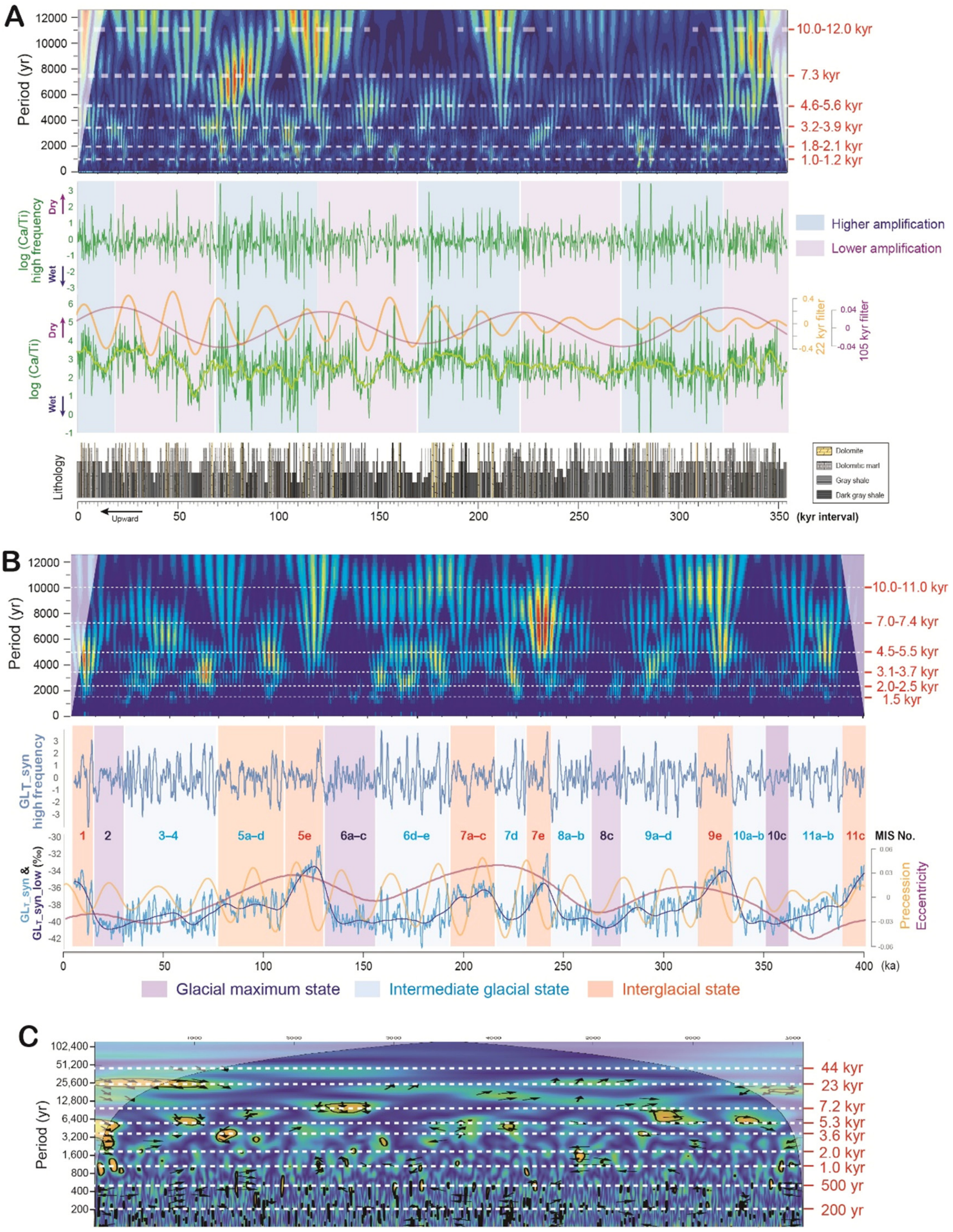

Fig. S13. Comparison of millennial-scale variation patterns between the Cretaceous lacustrine deposits and the late Pleistocene Greenland temperature record. (A) Lithology, Ca/Ti ratios and wavelet power spectrum for a ~355-kyr interval of core CSH01. **(B)** Greenland temperature variability record (GL$_T$_syn_high)[7] of the last 400 kyrs and wavelet power spectrum. **(C)** Wavelet coherence analysis of Ca/Ti ratio in Cretaceous lacustrine deposits and Greenland temperature variability record for ~355-kyr interval.

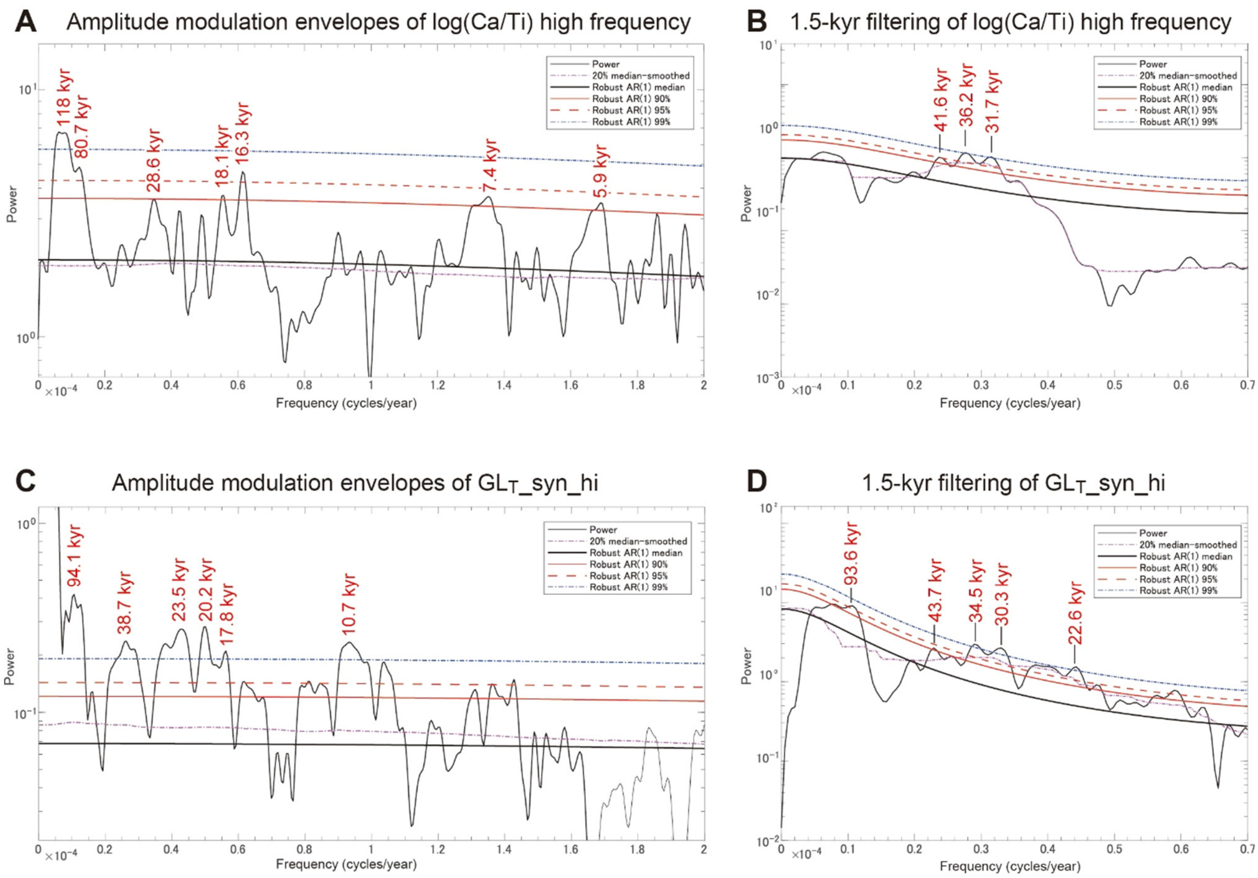

Fig. S14. MTM power spectrum of (A) AM envelopes of log(Ca/Ti) high frequency, (B) 1.5 kyr filtering of log(Ca/Ti) high frequency, (C) AM envelopes of GL$_{T}$_syn_hi, (B) 1.5 kyr filtering of GL$_{T}$_syn_hi (shown in Fig. 5).

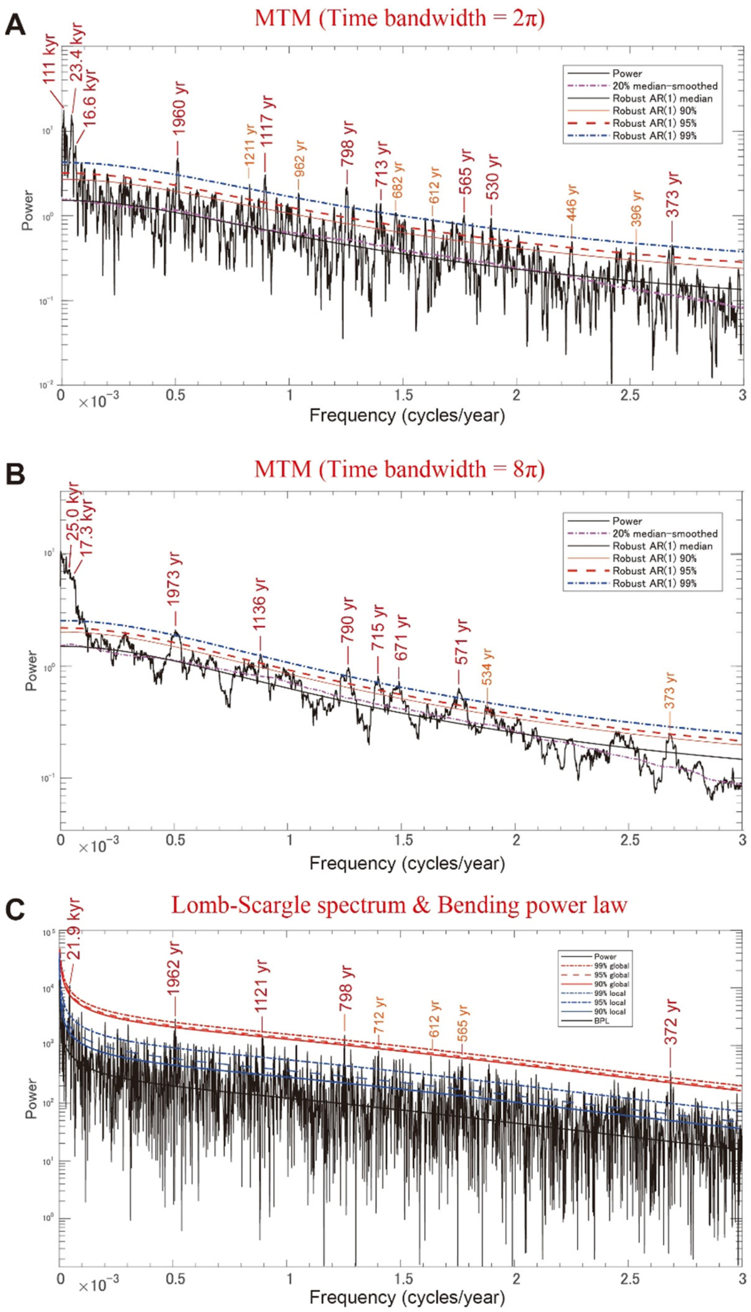

Fig. S15. Comparison between 2π MTM power spectrum (A), 8π MTM power spectrum (B), and Lomb-Scargle spectrum of Bending power law method (C) for sedimentation rate-calibrated log (Ca/Ti) data of CSH01 core. Reddish numbers indicate significant spectral peaks above 99 % CL, while yellowish numbers indicate moderate spectral peaks. Although fewer spectral peaks are detected in 8π MTM spectrum (**B**) and Bending power law method (**C**) compared to those of 2π MTM spectrum (**A**), the spectral peaks which significantly exceeding above 99 % CL in 2π MTM spectrum (reddish numbers) are rather consistent with that of Bending power law method.

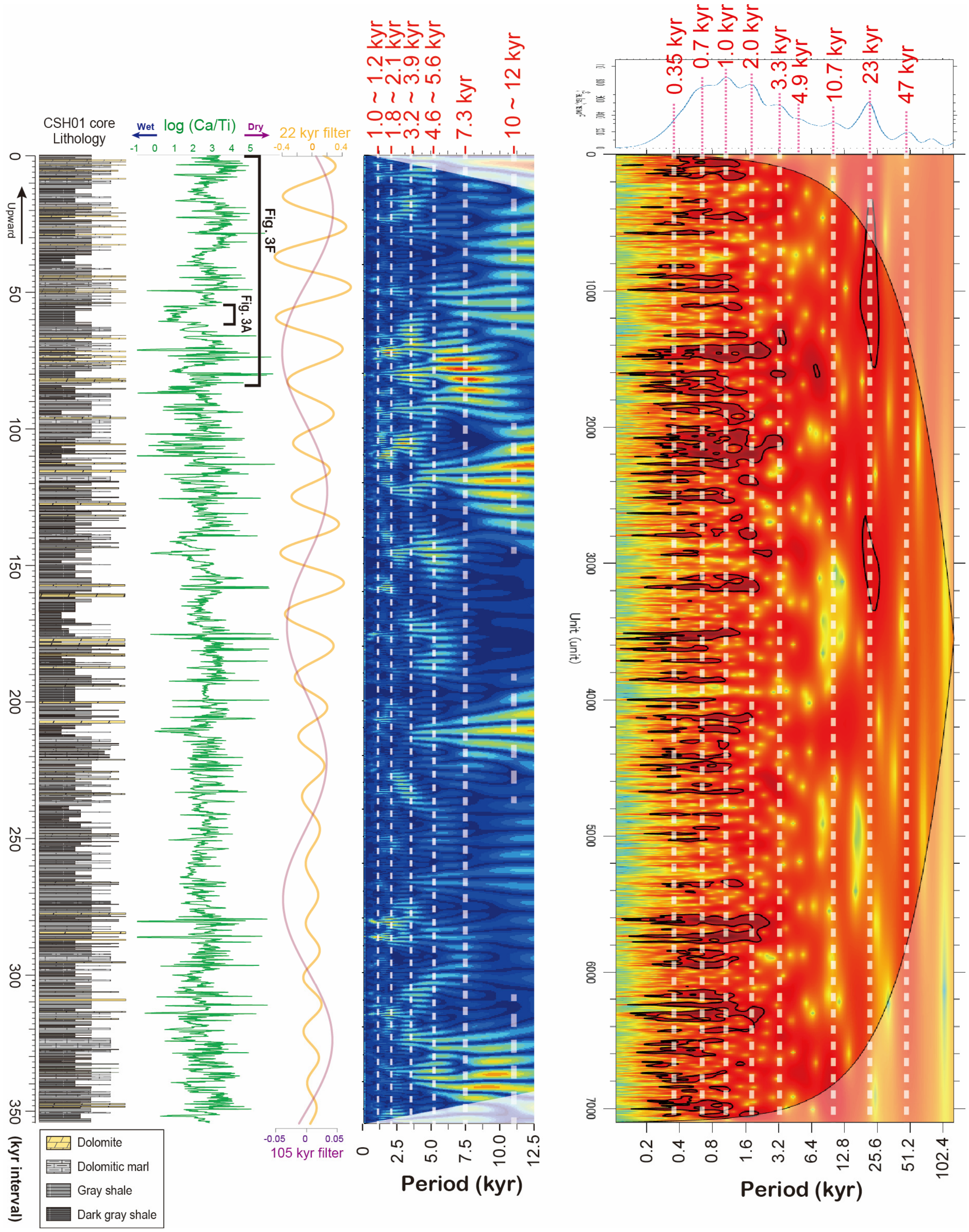

Fig. S16. Comparison between our wavelet analysis (using the Morlet wavelet function of Scilab software; shown in Fig. 4A) and wavelet analysis using Matlab software. Although the obtained periodicities are rather consistent, millennial-scale periodicities are more clearly distinguished in our wavelet analysis.

Table S1. Location, lake depth, varve sediment type and reference cited in **Fig. S7**.

	**Lake site**	**Country**	**Latitude**	**Longitude**	**Altitude**	**Depth**	**Varve sediment type**	**Ref.**
1	Kassjön	Sweden	63°55'N	20°01'E	84 m	12.2 m	Organic-clastic varve	*97*
2	Nylandssjön	Sweden	65°27'N	18°17'E	34 m	17.5 m	Organic-clastic varve	*98*
3	Sagtjernet	Norway	60°54'N	11°34'E	180 m	12 m	Organic-clastic varve	*99*
4	Kälksjön	Sweden	60°09'N	13°03'E	98 m	14.3 m	Organic-clastic varve	*100*
5	Furskogstjärnet	Sweden	59°23'N	12°05'E	137 m	14.2 m	Organic-clastic varve	*101*
6	Sarsjön	Sweden	64°02'N	19°36'E	177 m	7.3 m	Organic-clastic varve	*102*
7	Alimmainen Savijärvi	Finland	61°45'N	24°24'E	116 m	10.8 m	Organic-clastic varve	*103,104*
8	Nautajärvi	Finland	61°48'N	24°41'E	104 m	20 m	Organic-clastic varve	*104,105*
9	Lehmilampi	Finland	63°37'N	29°06'E	96 m	10.8 m	Organic-clastic varve	*106*
10	Lovojärvi	Finland	61°04'N	25°03'E	108 m	17.5 m	Organic-clastic varve	*107*
11	Rouge Tougjärv	Estonia	57°44'N	26°54'E	107 m	17.5 m	Clastic varve	*108*
12	Szurpiły	Poland	54°14'N	22°54'E	183 m	46.2 m	Organic-calcite varve	*109*
13	Lazduny	Poland	53°51'N	21°57'E	129 m	22.4 m	Organic-calcite varve	*110*
14	Lubinskie	Poland	52°18'N	14°54'E	71 m	21.6 m	Organic-calcite varve	*99,111*
15	Ammersee	Germany	48°00'N	11°07'E	533 m	81.1 m	Organic-calcite varve	*112*
16	Sacrower See	Germany	52°27'N	13°06'E	30 m	36 m	Organic-calcite varve	*44,113,114*
17	Tiefer See	Germany	53°35'N	12°32'E	62 m	62 m	Organic-calcite varve	*45,115,116*
18	Belau	Germany	54°06'N	10°16'E	29 m	29 m	Organic-calcite varve	*117*
19	Holzmaar	Germany	50°07'N	06°53'E	425 m	20 m	Organic-clastic varve	*118,119*
20	Meerfelder Maar	Germany	50°06'N	06°45'E	337 m	18 m	Organic-clastic varve (Siderite in late Allerød)	*50,120,121*
21	Diss Mere	England	52°22'N	01°06'E	26 m	6 m	Evaporitic varve (calcite)	*122*
22	Loe Pool	England	50°40'N	05°17'E	4 m	8 m	Organic-clastic varve	*123*
23	Greifensee	Switzerland	47°21'N	08°40'E	435 m	32 m	Organic-calcite varve	*124*
24	Baldeggerse	Switzerland	47°10'N	08°17'E	463 m	33 m	Organic-calcite varve	*125*
25	Soppensee	Switzerland	47°05'N	08°05'E	596 m	27 m	Organic-calcite varve	*126*
26	Oeschine	Switzerland	46°30'N	07°44'E	1580 m	56 m	Organic-calcite varve	*127*
27	Brienz	Switzerland	46°45'N	08°01'E	564 m	260 m	Organic-clastic varve	*128*
28	Silvaplana	Switzerland	46°27'N	09°48'E	1800 m	78 m	Organic-clastic varve	*129*
29	Seebergsee	Switzerland	46°37'N	07°28'E	1830 m	26 m	Organic-clastic varve	*130*
30	Bourget	France	45°45'N	05°51'E	234 m	145 m	Organic-calcite varve	*131*
31	Lac Pavin	France	45°55'N	02°54'E	1197 m	92 m	Organic-siderite varve	*132*
32	Bramant	France	45°70'N	06°60'E	2448 m	39 m	Organic-clastic varve	*133*
33	Lago di Ledro	Italy	45°52'N	10°45'E	653 m	46 m	Organic-calcite varve	*134*
34	Lago di Mezzano	Italy	42°37'N	11°56'E	452 m	31 m	Organic-clastic varve	*135*
35	Lago Grande di Monticchio	Italy	40°56'N	15°35'E	656 m	36 m	Organic-calcite varve	*136,137*
36	Balaton	Hungary	46°50'N	17°44'E	105 m	12.2 m	Dolomite	*41,138*
37	Neusiedler	Austria	47°50'N	16°45'E	115 m	1.8 m	Dolomite	*41,139*
38	Butrint	Albania	39°47'N	20°01'E	0 m	12 m	Evaporitic varve (aragonite)	*140*
39	Specchio di Venere	Pantelleria island, Italy	36°49'N	11°59'E	0 m	12.5 m	Dolomite	*41,141*
40	Salines	Spain	38°30'N	0°53'E	475 m	0 m	Dolomite	*41,42,142*
41	La Cruz	Spain	39°59'N	1°52'E	1000 m	21 m	Dolomite, Evaporitic varve	*143*
42	Zoñar	Spain	37°29'N	4°41'E	300 m	14.5 m	Evaporitic varve (calcite)	*144*
43	Montcortés	Spain	42°19'N	00°59'E	1027 m	30 m	Organic-calcite varve	*145*
44	Tuz	Turkey	38°45'N	33°21'E	905 m	1.5 m	Dolomite	*41,146*
45	Nar	Turkey	38°22'N	34°27'E	1363 m	26 m	Organic-calcite varve	*147,148*
46	Van	Turkey	38°32'N	43°00'E	1648 m	460 m	Organic-calcite varve	*96,149*
47	Urmia	Iran	37°42'N	45°19'E	1250 m	16 m	Dolomite	*41,150*
48	Dead Sea	Israel	31°20'N	35°30'E	-423 m	298 m	Evaporitic varve (aragonite)	*82,151*
49	Yoa	Chad	19°03'N	20°31'E	380 m	26 m	Organic-calcite varve	*152,153*
50	Botsumtwi	Ghana	06°30'N	01°25'E	99 m	81 m	Organic-calcite varve	*154*
51	Dasht-i Nawur	Afghanistan	33°34'N	67°46'E	3125 m	0.5 m	Dolomite	*41*
52	Didwana	India	27°23'N	74°34'E	336 m	5 m	Dolomite	*41,155*
53	Balkhash	Kazakhstan	46°10'N	74°20'E	341 m	24 m	Dolomite	*156,157*

54	Sary Chelek	Kyrgyzstan	41°54'N	71°57'E	1873 m	213 m	Organic-calcite varve	*158*
55	Chatyr Kol	Kyrgyzstan	40°37'N	75°18'E	3530 m	20 m	Organic-calcite varve	*159*
56	Sugan	China	38°52'N	93°54'E	2793 m	6 m	Evaporitic varve (aragonite)	*160*
57	Teletskoye	Russia	51°35'N	87°41'E	434 m	330 m	Organic-clastic varve	*161*
58	Shira	Russia	54°30'N	90°11'E	352 m	24 m	Evaporitic varve (calcite)	*162*
59	Bayan	Mongolia	50°00'N	94°00'E	932 m	29.2 m	Evaporitic varve (aragonite)	*163*
60	Telmen	Mongolia	48°48'N	97°20'E	1789 m	25 m	Organic-calcite varve	*164*
61	Sangiin Dalai	Mongolia	49°16'N	98°56'E	1988 m	26 m	Organic-calcite varve	*165*
62	Xinluhai	China	31°49'N	99°06'E	4020 m	65 m	Clastic varve	*166*
63	Twintaung	Myanmar	22°22'N	95°02'E	385	50 m	Organic-calcite varve	*167*
64	Huguang Maar	China	21°09'N	110°17'E	-154 m	22 m	Organic-siderite varve	*168,169*
65	Angulinuo	China	41°21'N	114°23'E	1312 m	6 m	Clastic varve	*170*
66	Xiaolongwan	China	42°18'N	126°21'E	655 m	15 m	Organic-clastic varve	*171*
67	Tougou-ike	Japan	35°29'N	133°55'E	0 m	5 m	Organic-siderite varve	*172*
68	Suigetsu	Japan	35°35'N	135°53'E	0 m	34 m	Organic-siderite varve	*38,93,173*

Supplementary References

97. G. Petterson, *et al.*, *Journal of Paleolimnology 22*, 443–455 (2010).
98. V. Gälman, *et al.*, *Journal of Paleolimnology 35*, 837–853 (2006).
99. B. Zolitschka, ed., *3rd Workshop of the PAGES Varves Working Group,* 110p. (2012).
100. T. Stanton, *et al.*, *Quaternary Geochronology 5*, 611–624 (2010).
101. L. M. Zillén, *et al.*, *Quaternary Science Reviews 21*, 1583–1591 (2002).
102. I. Snowball, *et al.*, *The Holocene 9*, 353–362 (1999).
103. A. E. K. Ojala, *et al.*, *Boreal Environment Reseach 5*, 243–255 (2000).
104. A. E. K. Ojala, M. Tiljander, *Quaternary Science Reviews 22*, 1787–1803 (2003).
105. A. E. K. Ojala, T. Alenius, *Palaeogeography, Palaeoclimatology, Palaeoecology 219*, 285–302 (2005).
106. E. Haltia-Hovi, *et al.*, *Quaternary Science Reviews 26*, 678–689 (2007).
107. M. Saarnisto, *et al.*, *Ann. Bot. Fennici 14*, 35–45 (1977).
108. S. Veski, *et al.*, *Geology 32*, 681–684 (2004).
109. M. Kinder, *et al.*, *GFF 135*, 248–257 (2013).
110. W. Tylmann, *et al.*, *Quaternary Geochronology 15*, 98–107 (2013).
111. M. Zielinski, *et al.*, *Water 11*, 2231; doi:10.3390/w11112231 (2019).
112. M. Czymzik, *et al.*, *Water Resources Research 46*, W11528, doi:10.1029/2009WR008360 (2010).
113. B. Lüder, *et al.*, *Journal of Paleolimnology*, 35, 897–912 (2006).
114. P. Bluszcz, *et al.*, *Journal of Limnology*, 68, 257–273 (2009).
115. U. Kienel, *et al.*, *J Paleolimnol 50*, 535–544 (2013).
116. N. Dräger, *et al.*, *Journal of Paleolimnology*, 62, 181–194 (2019).
117. W. Dörfler, *et al.*, *The Holocene 22*, 1413–1426 (2012).
118. B. Zolitschka, *et al.*, *Geology 28*, 783–786 (2000).
119. U. Kienel, *et al.*, *J Paleolimnol 33*, 327–347 (2005).
120. A. Lücke, A. Brauer, *Palaeogeography, Palaeoclimatology, Paleoecology*, 211, 139–155 (2004).
121. C. Martín-Puertas, C., *et al.*, *Journal of Quaternary Science*, *32*, 427–436 (2017).
122. S. M. Peglar, *et al.*, *Boreas 13*, 13–28 (1984).
123. H. Simola, *et al.*, *Nature 290*, 38–241 (1981).
124. D. M. Imboden, S. Emerson, *Limnology and Oceanography 23*, 77–90 (1978).
125. A. F. Lotter, *et al.*, *Journal of Paleolimnology 18*, 395–420 (1997).
126. A. F. Lotter, *Journal of Paleolimnology 25*, 65–79 (2001).
127. B. Amann, *et al.*, *Journal of Paleolimnology 51*, 375–391 (2014).
128. M. Sturm, A. Matter, *Spec. Publs. int. Ass. Sediment. 2*, 147–168 (1978).

129. A. Blass, *et al.*, *Quaternary Research 68*, 184–195 (2007).
130. S. Hausmann, *et al.*, *The Holocene 12*, 279–289 (2002).
131. C. Giguet-Covex, *et al.*, *Journal of Paleolimnology 43*, 171–190 (2010).
132. G. Schettler *et al.*, *Chemical Geology 240*, 11–35 (2007).
133. H. Guyard, *et al.*, *Quaternary Science Reviews 26*, 2644–2660 (2007).
134. S. B. Wirth, *et al.*, *Geophysical Research Letters 40*, 4025–4029 (2013).
135. A. Ramrath, *et al.*, *Journal of Paleolimnology 21*, 423–435 (1999).
136. B. Zolitschka, J. F. W. Negendank, *Quaternary Science Reviews 15*, 101–112 (1996).
137. J. R. M. Allen *et al.*, *Quaternary International 88*, 69–80 (2002).
138. E. Tompa, *et al.*, *Central European Geology 57*, 113–136 (2014).
139. D. Fussmann *et al.*, *Biogeosciences Discussions*, doi.org/10.5194/bg-2019-449 (2019).
140. D. Ariztegui, *et al.*, *Global and Planetary Change 7*, 183–192 (2010).
141. M. Cangemi, *et al.*, *Applied geochemistry*, 67, 168–176 (2016).
142. I. Queralt, *et al.*, *American Mineralogist*, 82, 812–819 (1997).
143. L. Romero-Viana, *et al.*, *Journal of Paleolimnology*, 40, 703–714 (2008).
144. C. Martín-Puertas, C., *et al.*, *Quaternary Research* 71, 108–120 (2009).
145. J. P. Corella, *et al.*, *Journal of Paleolimnology 46*, 351–367 (2011).
146. O. Kilic, M. Kilic, *Scientific Research and Essays 5*, 1317–1324 (2010).
147. M. D. Jones, *et al.*, *Geology 34*, 361–364 (2006).
148. J. Woodbridge, N. Roberts, *Quaternary Science Reviews 30*, 3381–3392 (2011).
149. M. Stockhecke, *et al.*, *Sedimentology 61*, 1830–1861 (2014).
150. K. Kelts, M. Shahrabi, *Paleogeography, Paleoclimatology, Paleoecology 54*, 105–130 (1986).
151. I. Neugebauer, *et al.*, *The Holocene 25*, 1358–1371 (2015).
152. S. Kröpelin, *et al.*, *Science 320*, 765–768 (2008).
153. P. Francus, *et al.*, *Sedimentology 60*, 911–934 (2013).
154. T. M. Shanahan, *et al.*, *Journal of Paleolimnology 40*, 339–355 (2008).
155. P. D. Roy, *et al.*, *Quaternary International 144*, 84–98 (2006).
156. T. Petr, *Int. J. Salt Lake Res. 1*, 21–46 (1992).
157. T. Chiba, *et al.*, *Quaternary International 397*, 330–341 (2016).
158. S. Lauterbach, *et al.*, *Quaternary Research 92*, 288–303 (2019).
159. J. Kalanke, *et al.*, *INQUA 2019*, abstract (2019).
160. A. Zhou, A., *et al.*, *Science in China Series D 50*, 1218–1224 (2007).
161. I. Kalugin, I., *et al.*, *Quaternary International 136*, 5–13 (2005).
162. I. Kalugin, I., *et al.*, *Quaternary International 290-291*, 245–252 (2013).
163. S. Naumann, *Die Erde 130*, 117–130 (1999).
164. J. A. Peck, *et al.*, *Paleogeography, Palaeoclimatology, Palaeoecology 183*, 135–153 (2002).
165. H. Hasegawa, *et al.*, in preparation.
166. G. Chu, *et al.*, *J. Geophys. Res. 116*, D02116, doi:10.1029/2010JD014454 (2011).
167. Q. Sun, *et al.*, *J. Geophys. Res., 121*, 5620–5630 (2015).
168. G. Chu, *et al.*, *Chinese Science Bulletin*, *45*, 2292–2295 (2000).
169. G. Yancheva, *et al.*, *Nature*, *445*, 74–77 (2007).
170. Q. Zhai, *et al.*, *Palaeogeography, Palaeoclimatology, Palaeoecology 241*, 95–102 (2006).
171. G. Chu, *et al.*, *Geophysical Research Letters 114*, D22108, doi:10.1029/2009JD012077 (2009).
172. M. Kato, *et al.*, *Quaternary International 105*, 33–37 (2003).
173. G. Schlolaut, *et al.*, *Quaternary Science Reviews*, *200*, 351–366 (2018).
